# CUT&Tag for High‐Resolution Epigenomic Profiling From a Low Amount of *Arabidopsis* Tissue

**DOI:** 10.1002/pld3.70161

**Published:** 2026-04-10

**Authors:** Yixuan Fu, Marc W. Schmid, Sara Simonini

**Affiliations:** ^1^ Institute of Plant and Microbial Biology University of Zurich Zurich Switzerland; ^2^ MWSchmid GmbH Glarus Switzerland

**Keywords:** *Arabidopsis*, CUT&Tag, epigenome profiling, epigenomics, histone modifications, next‐generation sequencing, nucleosomes

## Abstract

**Background:**

The genome‐wide profiling of chromatin states that are defined by different histone posttranslational modifications, known as epigenomic profiling, is crucial for understanding the epigenetic regulations of gene expression, both in animal and plant systems. CUT&Tag (Cleavage Under Targets and Tagmentation) is a novel enzyme‐tethering method for epigenomic profiling, initially developed for mammalian cells. CUT&Tag has several advantages compared to the most commonly used epigenomic profiling methods such as chromatin immunoprecipitation followed by high‐throughput sequencing (ChIP‐seq). CUT&Tag allows epigenenomic profiling from a much smaller amount of starting material compared to ChIP‐seq. Moreover, CUT&Tag relies on in situ DNA cleavage mediated by a transposase tethered to antibodies, while ChIP‐seq typically involves nearly random DNA fragmentation. This fundamental difference raises the important question of whether CUT&Tag provides distinct advantages in terms of resolution and the precision of epigenomic profiles compared to ChIP‐seq.

**Results:**

We profiled the genome‐wide distribution of three histone modifications, H3K27me3, H3K4me3, and H3K27Ac, from a few seedlings of *Arabidopsis* that weighed around 0.01 g. We compared the CUT&Tag H3K27me3 profile with publicly available H3K27me3 profiles generated from ChIP‐seq, and ChIPmentation, a ChIP‐seq–based technique where MNase and Tn5 transposon are used to cleave the chromatin and prepare sequencing libraries, respectively. We showed that all these techniques capture the same broad lines of the epigenomes, but they also showed preferences toward different genomic features and revealed different sets of peaks. Analysis using the CUT&Tag datasets for the three histone modifications revealed their genomic locations and their relationship with the gene expression level, which are consistent with the expected effect of these histone marks on gene transcription. By comparing to the nucleosome occupancy data, we show that CUT&Tag reached resolution at the single‐nucleosome level, which is similar to that of ChIPmentation, but higher than that of ChIP‐seq. Moreover, both CUT&Tag and ChIPmentation data revealed an enrichment of H3K27me3 mark on exons, thus providing a deeper understanding of epigenome features that could not be resolved by ChIP‐seq.

**Conclusion:**

CUT&Tag is a valid, easy‐to‐perform, cost‐effective, and reliable approach for efficient epigenomic profiling in *Arabidopsis*, compatible with limited amounts of plant tissue, and provides a higher resolution compared to that of ChIP‐seq. Because the CUT&Tag protocol starting input is isolated nuclei, it is applicable to model and nonmodel plants.

## 1 Background

The profiling of the genome‐wide occupancy of histone posttranslational modifications and the binding of transcription factors is crucial for understanding chromatin and epigenetic regulation in multicellular organisms. The most commonly used method for chromatin profiling is chromatin immunoprecipitation followed by high‐throughput sequencing (ChIP‐seq) (Yamaguchi et al. 2014; Kaufmann et al. 2010). In this approach, DNA‐protein interactions are preserved by crosslinking with formaldehyde, then the nuclei are extracted, chromatin is fragmented by sonication, and an antibody that targets the epitope of interest is added. Subsequently, the antibody‐bound chromatin is recovered, and DNA is purified, adapter‐ligated, amplified to generate a library, and sequenced through next‐generation sequencing. A major limitation in using this technique in plant cells is the requirement of grams of input material. This limit brings obstacles to the studies where only a small amount of input material is available, for example, the studies of the variation of epigenetic states between individual plants and the studies of epigenetic states in specific tissues that are small and hard to collect, like embryos and meristems.

**FIGURE 1:**
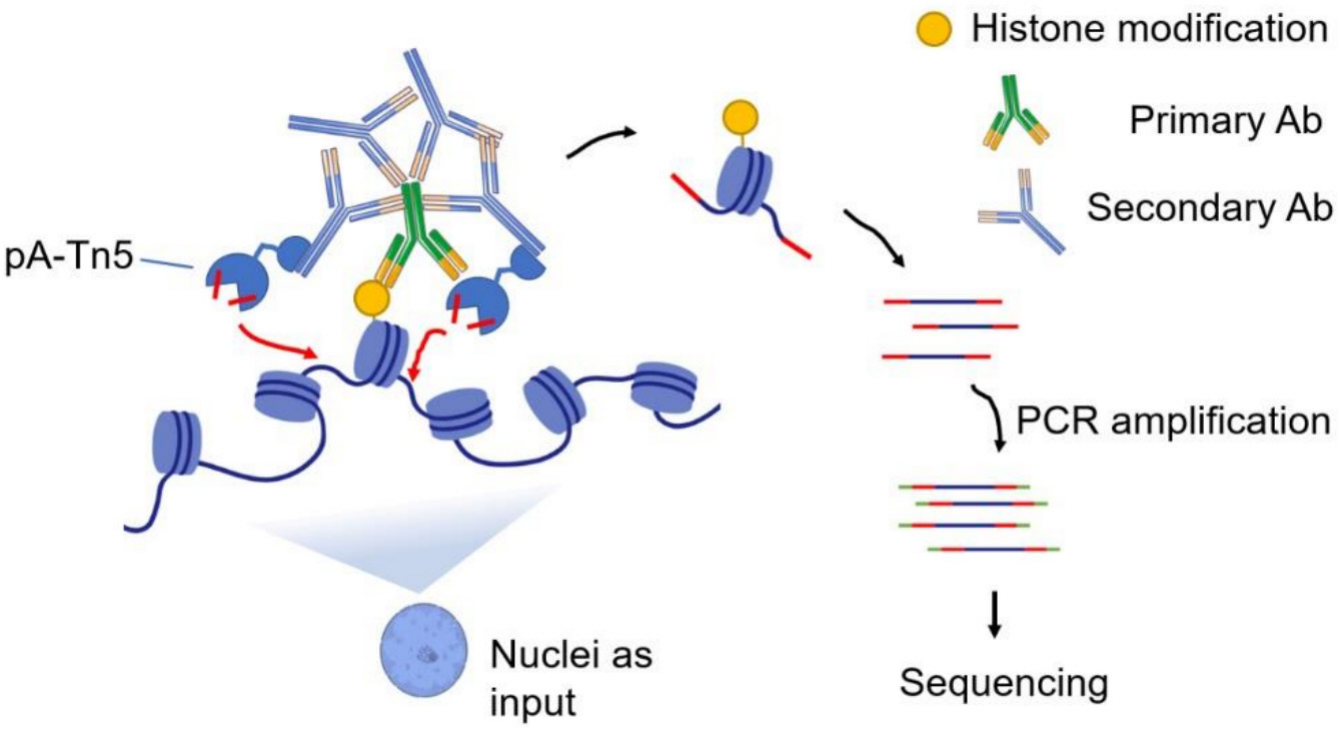
**Graphical summary showing the workflow of CUT&Tag using nuclei as input.** The nuclei are incubated with a primary antibody that binds to the histone modification of interest. Then, a secondary antibody tethers the pA‐Tn5 to the primary antibody. The pA‐Tn5 is activated, cleaves and tagments the DNA in proximity. The resulting tagmented DNA is released, and amplified with adapter‐containing oligos by PCR, resulting in sequencing‐ready libraries.

People have developed several improved versions of ChIP‐seq to address its material limitations. These include ULI‐ChIP (Brind'Amour et al. 2015) and ChIPmentation (Schmidl et al. 2015). Among these improved versions of ChIP‐seq, ChIPmentation has gained some popularity and has been successfully applied to plant systems (Schmidl et al. 2015; Zhu et al. 2023) ChIPmentation uses the same workflow as ChIP‐seq, but after the immunoprecipitation step, a Tn5 transposase is added to add adaptors to the ends of the DNA around the targeted nucleosomes. Primers binding to the adaptors are then used to amplify the tagmented fragments through PCR reactions. ChIPmentation has a reduced material requirement to a magnitude of 10^5^ cells. However, these improved versions of ChIP‐seq still follow the same strategy as the original ChIP‐seq and thus are similarly affected by technical issues, such as the limit of the fragmentation step, which causes material loss and a genome‐wide background (Skene and Henikoff 2017). A recently developed method based on the enzyme‐tethering strategy, CUT&Tag (short for Cleavage Under Targets and Tagmentation) (Kaya‐Okur et al. 2019), provides another valid alternative solution for chromatin profiling. CUT&Tag uses unfixed or lightly fixed cells or nuclei as input and a primary antibody to recognize the epitopes of interest, for example, histone posttranslational modifications, followed by a secondary antibody for signal amplification. Subsequently, a hyperactive Tn5 transposase‐protein A (pA‐Tn5) fusion protein loaded with DNA adaptors is added. The protein A part of pA‐Tn5 binds to antibodies with high affinity, thus tethering the pA‐Tn5 enzyme to the epitope of interest. After activation by magnesium, the hyperactive Tn5 transposase cleaves local genomic DNA and adds the DNA adapters to the cleaved ends in a process called tagmentation. After DNA extraction, the tagmented and adapter‐ligated DNA is amplified by PCR with full‐length oligos containing the entire sequencing adaptors, which results in sequencing‐ready libraries (Figure 1).

Compared to ChIP‐seq and its variants, CUT&Tag shows better compatibility with extremely low‐input materials in animal systems. It has been used with less than 100 mammalian cells, providing high‐quality datasets for several histone modifications (Kaya‐Okur et al. 2019; Kaya‐Okur et al. 2020). It was also successfully applied to systems with material limitations, as for example, mammalian embryos at very early stages of development (Gassler et al. 2022).

CUT&Tag represents one of the latest advancements in enzyme‐tethering epigenomic profiling techniques. Another popular enzyme‐tethering technique is CUT&RUN sequencing (Cleavage Under Targets and Release Using Nuclease) (Skene and Henikoff 2017), which works in a similar way to CUT&Tag: instead of pA‐Tn5, pA‐MNase is used to cleave and release the DNA fragments. Compared to CUT&RUN sequencing, the library preparation for CUT&Tag is more straightforward, where only PCR amplification is involved. In contrast, CUT&RUN requires additional ligation‐based library preparation.

Besides its compatibility with small amounts of material, CUT&Tag holds the potential to provide a higher resolution compared to ChIP‐seq. In ChIP‐seq, the chromatin is shared into 200–300 bp fragments (Kidder et al. 2011). Therefore, the ends of sequenced fragments may be up to hundreds of base pairs away from the position of the actual target. Differently, in CUT&Tag the transposase is tethered to the target through specific antibodies, thus making the DNA cleavage site in the near proximity of the target. This difference in the distance between the DNA cleavage site and the target might allow CUT&Tag to profile the location of the target most accurately and therefore to lead to higher resolution.

Moreover, many of these studies rely on isolated nuclei or purified cell populations as starting material, often using several thousand nuclei per reaction. This leaves open the question of whether CUT&Tag can provide a broadly applicable alternative for addressing developmental questions in plant research, where starting material is frequently limiting. Furthermore, there is currently no widely established benchmark or detailed CUT&Tag protocol demonstrating robust performance with different antibodies directly in Arabidopsis tissue.

CUT&Tag and CUT&RUN have gained considerable popularity in animal research and have recently been adapted to several plant systems, including cotton, rice, rapeseed, and Arabidopsis, for profiling histone modifications such as H3K4me3, H3K9me2, H3K9ac, and H3K27me3 (Huang and Sun 2022; Tao et al. 2022; Tao et al. 2023; Tao et al. 2020; Zhao et al. 2023; Ouyang et al. 2021; Zheng and Gehring 2019; Wu et al. 2022; Ouyang and Li 2023; Ouyang et al. 2022; Xue et al. 2024; Del Toro‐De León et al. 2024; Li et al. 2026; Qian et al. 2025). However, systematic evaluation of these approaches in plant systems remains limited, raising questions about their ability to reproduce ChIP‐seq results and whether they can provide improved resolution. Moreover, many of these studies rely on isolated nuclei or purified cell populations as starting material, often using several thousand nuclei per reaction. This leaves open the question of whether CUT&Tag can provide a broadly applicable alternative for addressing developmental questions in plant research, where starting material is frequently limiting. Furthermore, there is currently no widely established benchmark or detailed CUT&Tag protocol demonstrating robust performance with different antibodies directly in *Arabidopsis* tissue.

Here, we present an optimized, easy‐to‐perform, and reliable CUT&Tag protocol adapted to successfully work with *Arabidopsis thaliana* tissues. We tested our protocol with a minimum amount of seedling material (0.01 g) for three different histone modifications: H3K27me3, H3K4me3, and H3K27Ac, and used histone H3 as a control. By comparing CUT&Tag with ChIP‐seq datasets, we show that CUT&Tag, ChIP‐seq, and ChIPmentation capture the same broad lines of the epigenome, while they also capture sets of different peaks. Compared to ChIP‐seq, CUT&Tag shows higher sensitivity toward gene bodies, particularly exons. Moreover, we show that CUT&Tag has a resolution that reaches the single‐nucleosomal level, which is similar to ChIPmentation but is higher than ChIP‐seq. The increased resolution of CUT&Tag allows revealing the detailed patterns of histone modifications occupancy that were hard to decipher in ChIP‐seq data, including the enrichment of H3K27me3 at exons and the epigenetic states of single nucleosomes. These findings prove that CUT&Tag is a valid technique for epigenome profiling in plant tissues that allows for high resolution even with little input material.

## 2 Results

### 2.1 CUT&Tag Reveals Similar but Also Different Sets of H3K27me3 Peaks Compared to ChIP‐seq

We adapted and optimized the CUT&Tag protocol, originally developed for mammalian cells, for nuclei extracted from *Arabidopsis* seedlings and tested antibodies from various brands (Table 1). We noticed that the concentration of libraries generated by CUT&Tag can vary significantly depending on the antibody used. Notably, some antibodies effective in ChIP‐seq may not be compatible with CUT&Tag (Supplementary Figure S1).

**TABLE 1:** List of the antibodies tested by us and CUT&Tag approved.

Target	Brand	Catalogue number	Specie
H3K27me3	Cell Signaling Technology	9733	Rabbit
H3K4me3	Diagenode	c15410003	Rabbit
H3K27Ac	Abcam	4729	Rabbit
H3	Active Motif	39064	Mouse
Rabbit IgG	Antibodies‐online	ABIN101961	Guinea Pig
H2AUb	Cell Signaling Technology	8240	Rabbit
H3K4me2	Abcam	Ab32356	Rabbit

**FIGURE 2:**
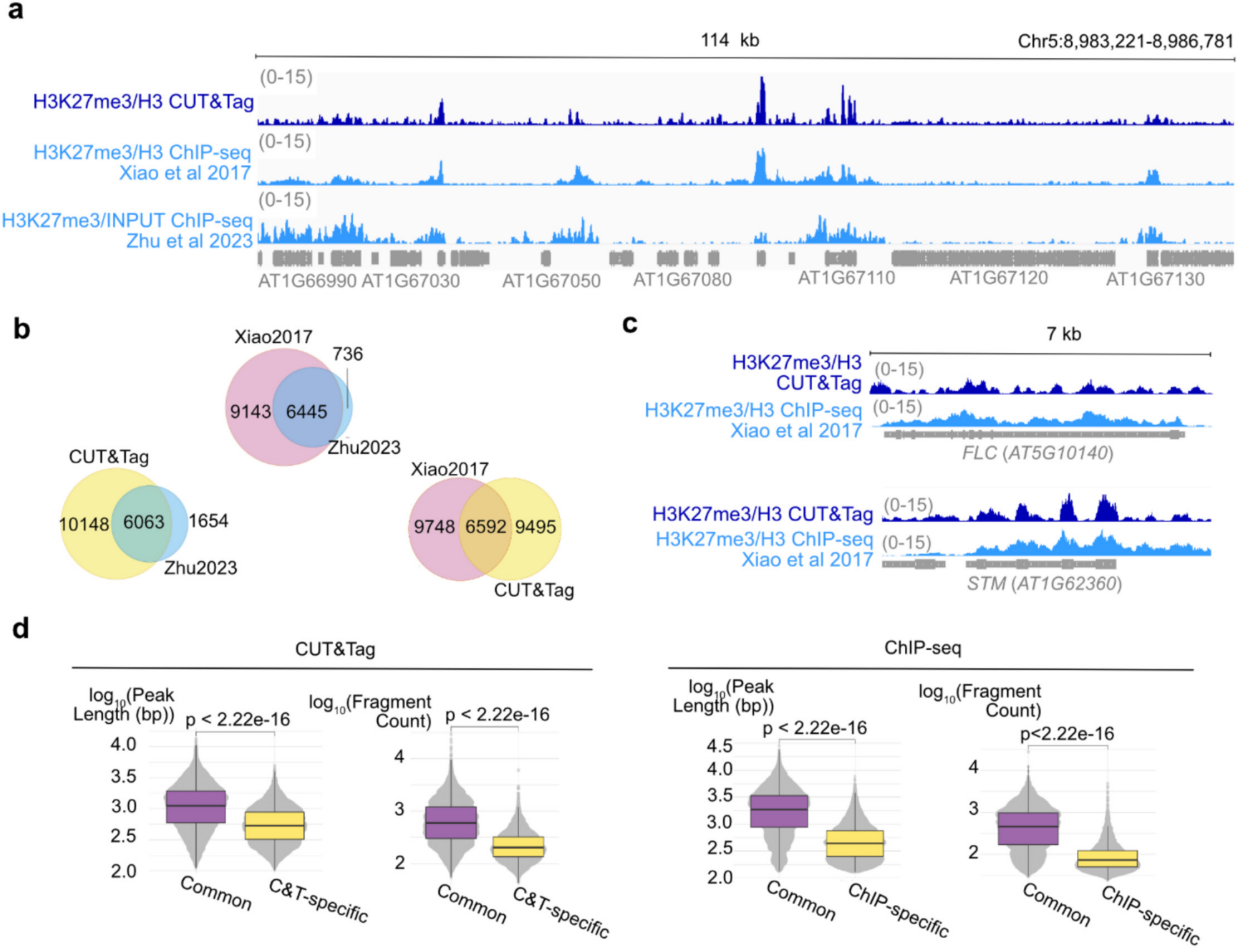
**Comparison between ChIP‐seq and CUT&Tag.** (a) An overview of a 114‐kb genomic window, showing the H3K27me3 data from CUT&Tag and two previously published ChIP‐seq datasets (Zhu et al. 2023; Xiao et al. 2017). The profiles show the visual similarity between the H3K27me3 from CUT&Tag and ChIP‐seq data from Xiao et al. (2017). H3K27me3 occupancy is normalized on H3 occupancy. (b) Venn diagram depicting the overlap between H3K27me3 peaks found by CUT&Tag (yellow circles) and a ChIP‐seq experiment (pink circles) and a ChIPmentation experiment (blue circles). (c) Browser views of H3K27me3 from CUT&Tag and ChIP‐seq at two PRC2‐target genes showing H3K27me3 enrichment at same locations. H3K27me3 occupancy is normalized on H3 occupancy. (d) Comparisons of the peak length and peak fragment count between peaks common (purple boxplot) in CUT&Tag (left side) and ChIP‐seq (right side), and peaks specific (yellow boxplot) to each method. Test method: Wilcoxon rank sum test; Sample size: CUT&Tag: 18516; ChIP‐seq: 16857.

We produced CUT&Tag datasets from *Arabidopsis* wild‐type seedlings for the histone modification H3K27me3 using histone H3 as controls. To assess the results of CUT&Tag, we compared our dataset with a publicly available ChIP‐seq dataset (Xiao et al. 2017) and a ChIPmentation dataset (Zhu et al. 2023) for the same histone modification. Starting from a few seedlings, we obtained around 8.7–15.3 million aligned, deduplicated fragments for each H3K27me3 CUT&Tag replicate (Supplementary Tables S1 and S2), which is considered good quality and sufficient for downstream analysis (Kaya‐Okur et al. 2019). The H3K27me3 profiles generated by our CUT&Tag analysis show enrichment patterns that typically span one or a few genes. We first noticed that the CUT&Tag H3K27me3 datasets lead to an H3K27me3 occupancy profile similar to the one obtained by ChIP‐seq performed by Xiao et al. (2017) (Figure 2a). Using the same method for peak calling with MACS2, the CUT&Tag H3K27me3 datasets revealed 18,517 peaks that were reproducible in at least two replicates, while 16,858 and 7842 peaks were detected by Xiao et al. and Zhu et al., respectively. The fewer peaks detected in Zhu et al. suggest that peaks captured by ChIPmentation are likely strong and stable target‐DNA interactions. CUT&Tag was able to capture nearly 80% of the ChIPmentation‐obtained peaks (Figure 2b), thus suggesting that CUT&Tag is suited to capture robust target‐DNA interactions.

Next, we focus on the comparison between our CUT&Tag datasets and the ChIP‐seq dataset from Xiao et al. (2017). While these two datasets share 6592 common peaks, 9748 peaks were specific to ChIP‐seq (referred to as ChIP‐specific peaks), and 9495 peaks were specific to CUT&Tag (referred to as C&T‐specific peaks).

Common peaks were found on genomic locations of some known *Polycomb* group proteins target genes, where strong enrichment of H3K27me3 is expected, for example *FLOWERING LOCUS C* (*FLC*) and *SHOOT MERISTEMLESS* (*STM*) (Figure 2c), which are characterized by long peaks (mean length equals 1463 bp for CUT&Tag peaks and 2575 bp for ChIP‐seq peaks, Figure 2d) with high numbers of fragment counts (mean fragment count per peak equal 941 for CUT&Tag peaks and 739 fragment counts per peak for ChIP‐seq peaks, Figure 2d). The C&T‐specific peaks and the ChIP‐specific peaks are characterized by short lengths (mean length equals 672 bp for C&T‐specific peaks and 627 bp for ChIP‐specific peaks) and fewer read counts than common peaks (Figure 2d), suggesting that these method‐specific peaks are associated with less predominant enrichment of H3K27me3.

We then question whether the H3K27me3 profile generated by CUT&Tag would be similar to CUT&RUN‐generated H3K27me3 profiles (Zheng and Gehring 2019) and found that the two profiles looked visually similar (Supplementary Figure S2). Next, we calculated the Pearson correlation coefficient and hierarchical clustering using one replicate from each method, including ChIP‐seq and ChIPmentation. All the H3K27me3 samples clustered together, and they shared Pearson correlation coefficients between 0.54 and 0.78 (Supplementary Figure S3). In contrast, all the controls (Histone H3 or INPUT) were clustered aside and showed lower Pearson correlation coefficients with the H3K27me3 datasets (Supplementary Figure S3). These results suggest that CUT&Tag and CUT&RUN, together with ChIP‐seq and ChIPmentation, generate similar profiles for H3K27me3.

### 2.2 CUT&Tag and ChIPmentation Show Higher Signals on Exons

**FIGURE 3:**
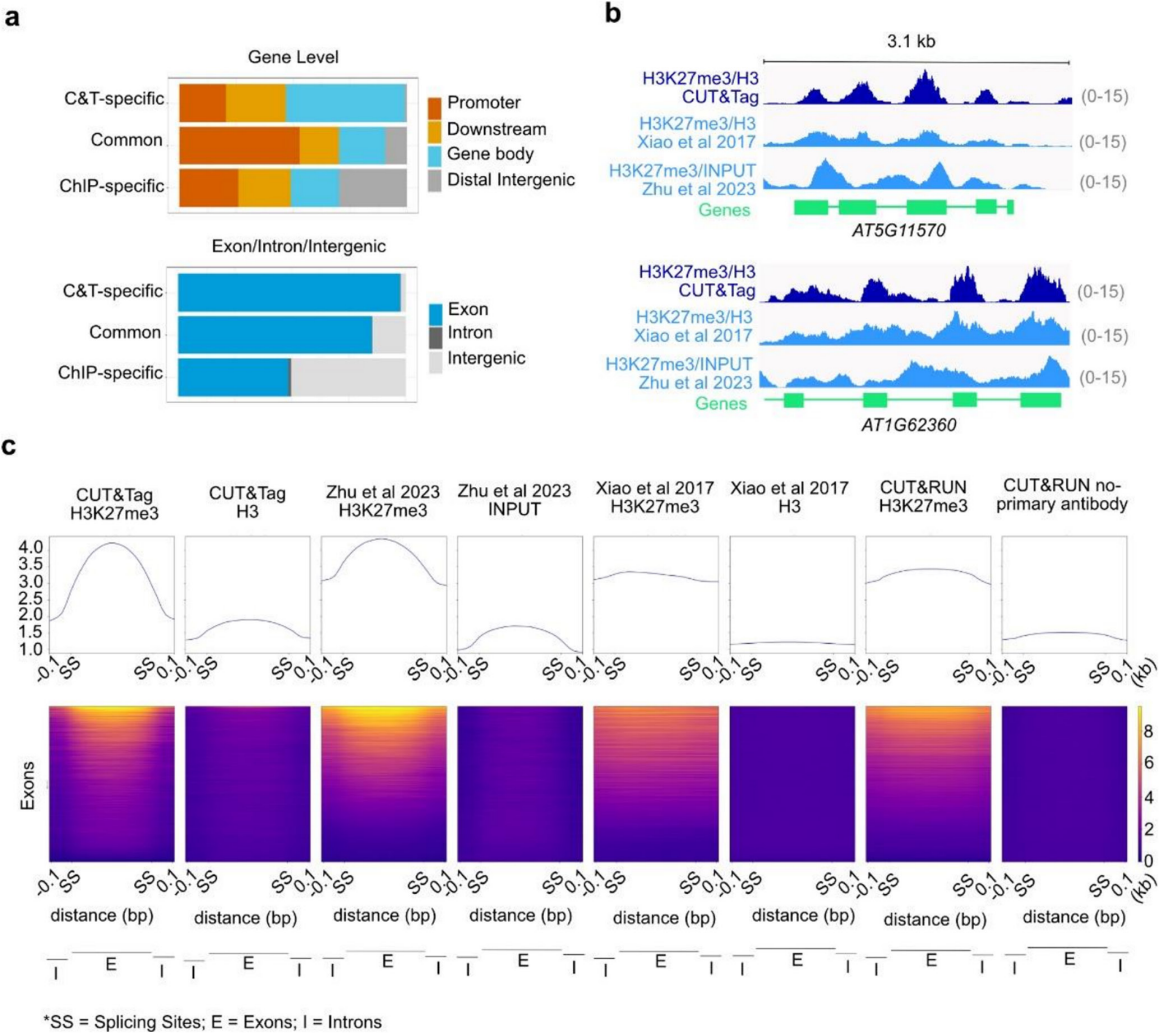
**Enrichment of H3K27me3 on exons profiled by CUT&Tag.** (a) Comparisons of genomic feature distribution of peaks common in ChIP‐seq and CUT&Tag, and peaks specific to each method. (b) Browser view of two example genes showing that CUT&Tag reveals strong enrichment of H3K27me3 on exons compared to introns, in comparison to ChIP‐Seq and ChIPmentation. H3K27me3 occupancy is normalized on H3 occupancy. (c) Heatplots showing the distribution of H3K27me3 and the H3 or INPUT controls signals (by CUT&Tag, ChIPmentation, ChIP‐Seq, and CUT&RUN) around exons. Only exons that overlap with H3K27me3 peaks found by at least one method are used for the heatmap. The first and the last exons are excluded; therefore, all the exons used here are flanked by introns on both sides. H3K27m3 occupancy is not normalized on H3 occupancy.

To better understand the origin of CUT&Tag and ChIP‐seq specific peaks, we plotted their genomic locations, along with the common peaks (Figure 3a). We found that, compared to the common peaks, C&T‐specific peaks are more likely located on gene bodies, particularly exons (two examples are shown in Supplementary Figure S4), while the ChIP‐specific peaks are particularly enriched in the intergenic regions (two examples shown in Supplementary Figure S4).

Consistent with our initial observation that CUT&Tag identifies more peaks on exons, we found that CUT&Tag H3K27me3 signal exhibits strong enrichment over exons compared to introns (Figure 3b). Heatmaps for both CUT&Tag H3K27me3 and H3 datasets confirm this trend, with H3K27me3 showing a notably higher degree of exon enrichment than H3. In contrast, ChIPmentation data (Zhu et al. 2023) show exon enrichment for both H3K27me3 and INPUT signals, suggesting that some enrichment may be inherent to the method. ChIP‐seq and CUT&RUN, however, show only mild but still detectable enrichment toward exons (Figure 3c).

Because exons generally have higher GC content than introns, we asked whether the observed exon‐biased signal could be attributed to GC content differences. To test this, we plotted the average CUT&Tag H3K27me3 signal across exons and introns matched for GC content, ranging from 30% to 70% (Supplementary Figure S5). At each GC level, signals on exons and introns were comparable, suggesting that GC content largely explains the observed difference—although we cannot fully exclude additional biological or technical factors.

Altogether, we showed that both CUT&Tag and ChIP‐seq capture genomic locations stably enriched in H3K27me3 and their specific signatures, thus demonstrating the reliability of CUT&Tag in performing well with *Arabidopsis* tissues. While both methods detect specific peak sets that are relatively short and weak—likely reflecting minor technical biases—we found a notable difference in exon‐level signal enrichment across techniques. Specifically, CUT&Tag and ChIPmentation show stronger enrichment of both H3K27me3 and control signals on exons compared to ChIP‐seq and CUT&RUN. This exon bias in CUT&Tag appears to be largely explained by GC content differences between exons and introns, as signal levels were comparable when GC content was matched. However, the stronger exon enrichment observed in CUT&Tag and ChIPmentation—but not in ChIP‐seq—remains an interesting difference, possibly reflecting their higher resolution and ability to profile histone modifications at the level of single nucleosomes. We explore this further in the final part of the results.

### 2.3 CUT&Tag Profiles of Three Histone Modifications in *Arabidopsis* Seedlings

**FIGURE 4:**
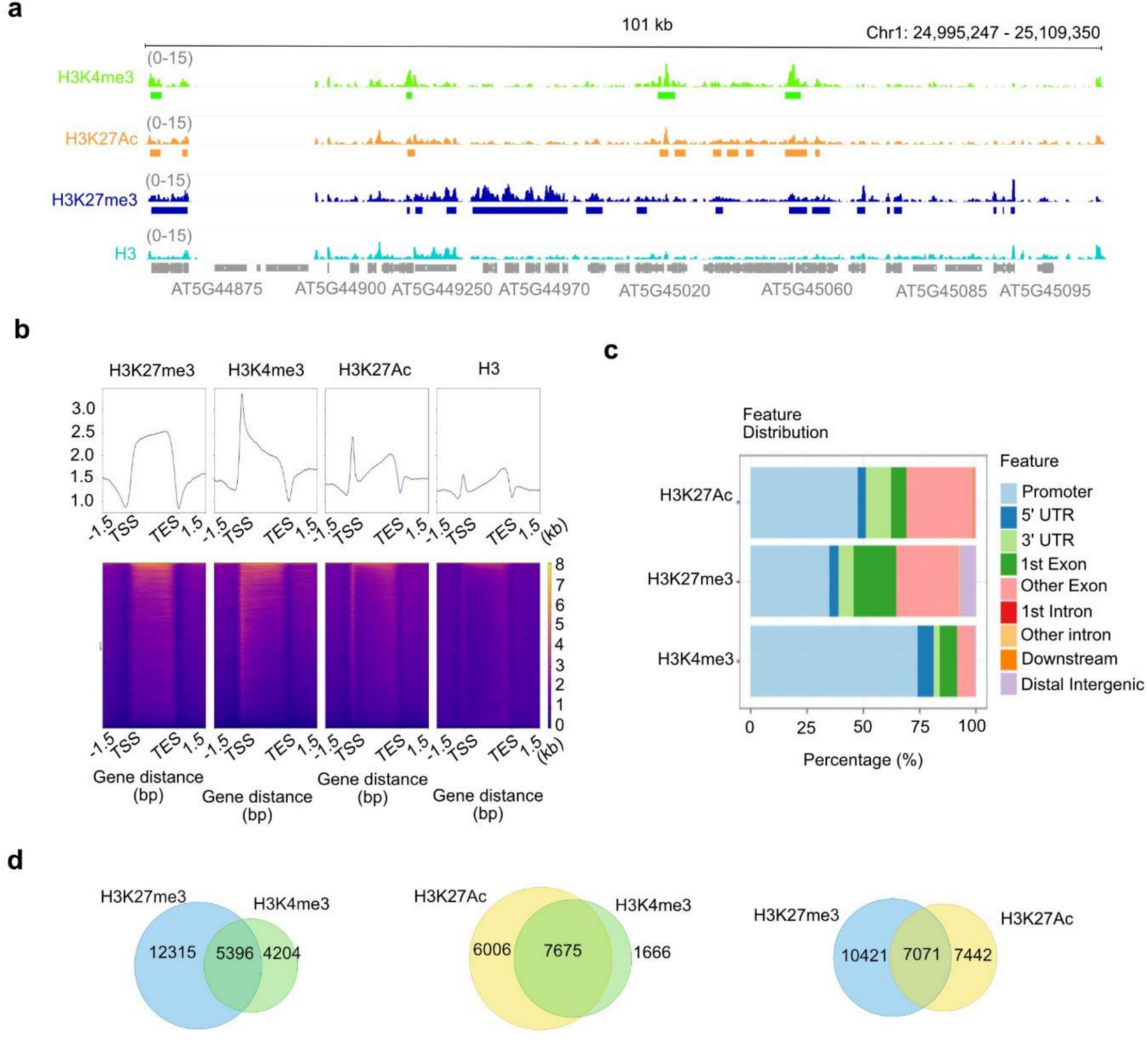
**The genome‐wide distribution of three histone modifications profiled by CUT&Tag.** (a) A browser view of 101‐kb genomic windows showing the profiles of three histone modifications and the H3 control profiled by CUT&Tag. The profiles show different features of peaks of the three histone modifications: H3K4me3 peaks are mostly located around transcription start sites, H3K27Ac peaks are located at promoters and gene bodies, and H3K27me3 peaks usually span one or a few genes. Histone modifications occupancy is not normalited on H3 occupancy, which is showed as separate track. (b) Heatplot showing the distribution of H3K27me3, H3K4me3, and H3K27Ac around the gene bodies. TSS, transcriptional start sites; TES, transcription end sites. (c) Genomic feature distribution of H3K27me3, H3K4me3, and H3K27Ac peaks. (d) Venn plots showing the pairwise overlap of CUT&Tag peaks of H3K27me3 (blue circle), H3K4me3 (green circle), and H3K27Ac (yellow circle).

Next, we employed CUT&Tag for profiling two additional histone modifications: H3K4me3 and H3K27Ac, which are two marks that correlate with active transcription. Figure 4a provides an overview of the enrichment of these three histone modifications compared to the H3 control, with the peaks indicated. Replicates for each histone modification correlate well and are clustered together (Supplementary Figure S6).

When comparing peak features such as length and location around genes (Figure 4b,c), we noticed that H3K27me3 signals are enriched on gene bodies, while H3K4me3 and H3K27Ac occupancy are particularly enriched around the transcriptional start site (both K4me3 and K27Ac) and gene bodies (mostly K27Ac). Notably, these observations are largely consistent with previous observations obtained by ChIP‐seq (Zhu et al. 2017; Yan et al. 2019).

We then compared the H3K27me3, H3K4me3, and H3K27Ac datasets and looked for common and different peaks (Figure 4d). As expected, H3K27Ac and H3K4me3 peaks shared a high degree of overlap, as they both have the same activating effect on transcription. Interestingly, we found that around half of the H3K27Ac and H3K4me3 peaks are also marked by H3K27me3. This degree of overlap is higher than the previously reported 15% overlap between H3K27me3 and H3K4me3 (Zhang et al. 2009).

### 2.4 CUT&Tag Reveals the Relations Between Histone Marks and Gene Expression

To understand if the epigenetic status revealed by CUT&Tag correlates with the transcriptional status of the peak‐bearing genes, we compared our CUT&Tag datasets with publicly available transcriptomic data (RNA‐Seq) from *Arabidopsis* seedlings. We observed that the presence of specific histone modifications correlates well with the expression levels of the targets detected in seedlings. For instance, floral and meristem identity genes such as *APETALA1* (*AP1*) and *STM* showed strong enrichment of H3K27me3 and depletion of H3K4me3 and H3K27Ac. Accordingly, both *STM* and *AP1* are repressed in seedlings by the activity of *Polycomb* group proteins (Zhu et al. 2023; Xiao et al. 2017; Kinoshita et al. 2001) (Supplementary Figure S7a). Actively transcribed genes, such as *RIBOSOMAL PROTEIN L7B* (*RPL7B*, *AT2G01250*) and *UBIQUITIN CARRIER PROTEIN 1* (*UBC1*, *AT1G14400*), exhibited enrichment of H3K4me3 and H3K27Ac (Supplementary Figure S7b).

**FIGURE 5:**
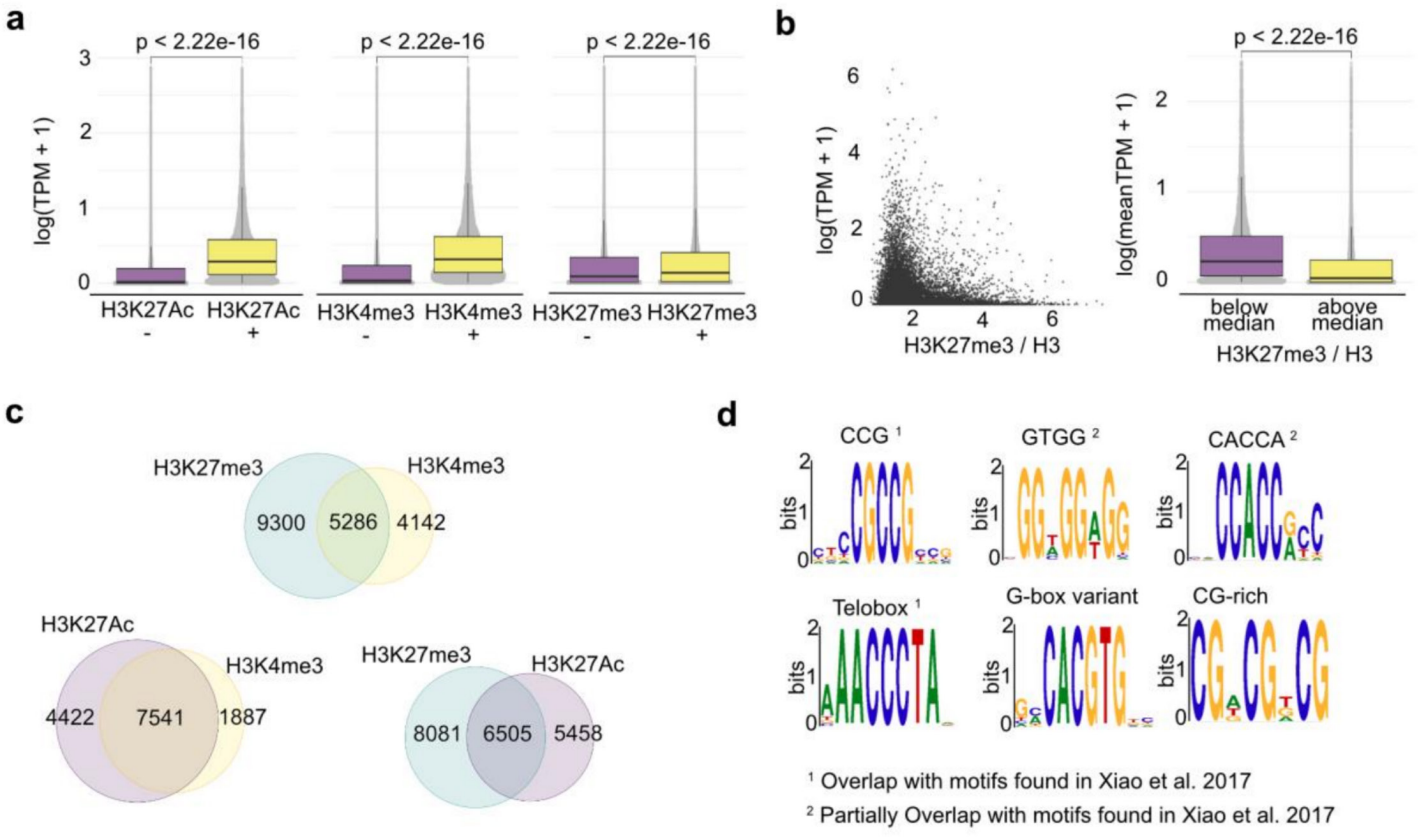
**Relation between histone marks and gene expression level, overlap between different histone marks, and motif enrichment analysis using the CUT&Tag datasets.** (a) Bar plots showing the comparison of the expression level between genes not marked (purple boxplot) and marked (yellow boxplot) by each histone modification. Test method: Wilcoxon rank sum test. Sample size: 32,106 for all three plots. (b) Dot plot and bar plot showing the anti‐correlation between the H3K27me3 level and expression among all H3K27me3‐marked genes. Test method: Wilcoxon rank sum test. Sample size: 14,586 (c) Venn diagram showing the pairwise overlap of genes marked by different histone marks H3K27me3 (blue circles), H3K4me3 (yellow circles), and H3K27Ac (purple circles). (d) Enriched motifs found from all replicates of H3K27me3 peaks; the motifs that are overlapped or partially overlapped with a previous report (Xiao et al. 2017) are labeled by 1 and 2.

Next, we plotted the expression levels of genes marked and not marked by each histone modification (Figure 5a), where a gene is classified as marked if at least one peak is detected in its promoter, 5′UTR, gene body, or 3′UTR regions. H3K4me3‐marked and H3K27Ac‐marked genes showed significantly higher expression than unmarked genes, which is consistent with the known association of these histone modifications with active transcription (Millán‐Zambrano et al. 2022). Interestingly, we found that H3K27me3‐marked genes had a slightly higher average expression level than unmarked genes, which is not the expected correlation, as H3K27me3 is a mark associated with gene repression (Cao et al. 2002). This could be explained by the fact that we used whole seedlings as starting material, which consist of various cell and tissue types. As a result, the dataset represents a mixture of occupancy from different cell types, where certain genes may be marked differently. Therefore, many of the H3K27me3 genes are likely only marked by H3K27me3 in a small population of cells but actively expressed in other cells. To further explore the relation between H3K27me3 and gene expression, we quantified the H3K27me3 enrichment level (H3K27me3/H3) and found that genes that are highly enriched in H3K27me3 tend to have low expression levels (Figure 5b). In agreement, we found that some genes normally expressed in discrete domains of the seedlings have both H3K27me3 and H3K4me3 marks. For instance, *TEOSINTE BRANCHED1/CYCLOIDEA/PCF 15* (*TCP15*, AT1G69690) was enriched in both H3K4me3/H3K27Ac and H3K27me3 modifications (Supplementary Figure S7c). TCP15 is normally expressed in young, proliferating leaves but silenced in older ones (Kieffer et al. 2011). Therefore, the detected mixed chromatin state may be the result of the sum of individual chromatin states, repressed and active, that depend on the cell type. This scenario became even more evident when comparing all three histone modifications against each other (Figure 5c). Indeed, we observed that around half of the H3K4me3/H3K27ac‐marked genes are also marked by H3K27me3.

In summary, we used CUT&Tag to map histone modification occupancy and coupled this with transcriptome analysis, providing biological insights into the effects of specific epigenetic states on gene transcription.

### 2.5 Motif Enrichment Analysis Using CUT&Tag Data Reveals Motifs Potentially Involved in H3K27me3 Deposition

Deposition of histone marks is made by histone‐modifying enzymes, which are recruited at genomic loci by the interaction with other proteins, or also by consensus sequences. In *Arabidopsis*, the recruitment of the *polycomb* repressive complex 2 (PRC2) is likely through interaction of the complex with transcription factors that bind to cis elements in the genome. Therefore, a motif search analysis of H3K27me3‐marked genomic fragments might help reveal cis elements that favor PRC2 recruitment (Xiao et al. 2017). The motif enrichment analysis on H3K27me3 CUT&Tag peaks revealed seven motifs (Figure 5d). Notably, four out of seven motifs overlap or partially overlap with motifs previously shown to be associated with PRC2 recruitment (Xiao et al. 2017), including, for instance, the Telobox. The other motifs include a G‐quadruplex–forming sequence (McQuaid et al. 2019) and a G‐box variant (Pérez‐Alonso et al. 2021).

To conclude, we explored DNA motif enrichment within the CUT&Tag datasets, which could aid in identifying chromatin regulators with roles in histone modification deposition/removal.

### 2.6 CUT&Tag and ChIPmentation Show Resolutions Close to Nucleosome Level, Which Is Higher Than Conventional ChIP‐seq

**FIGURE 6:**
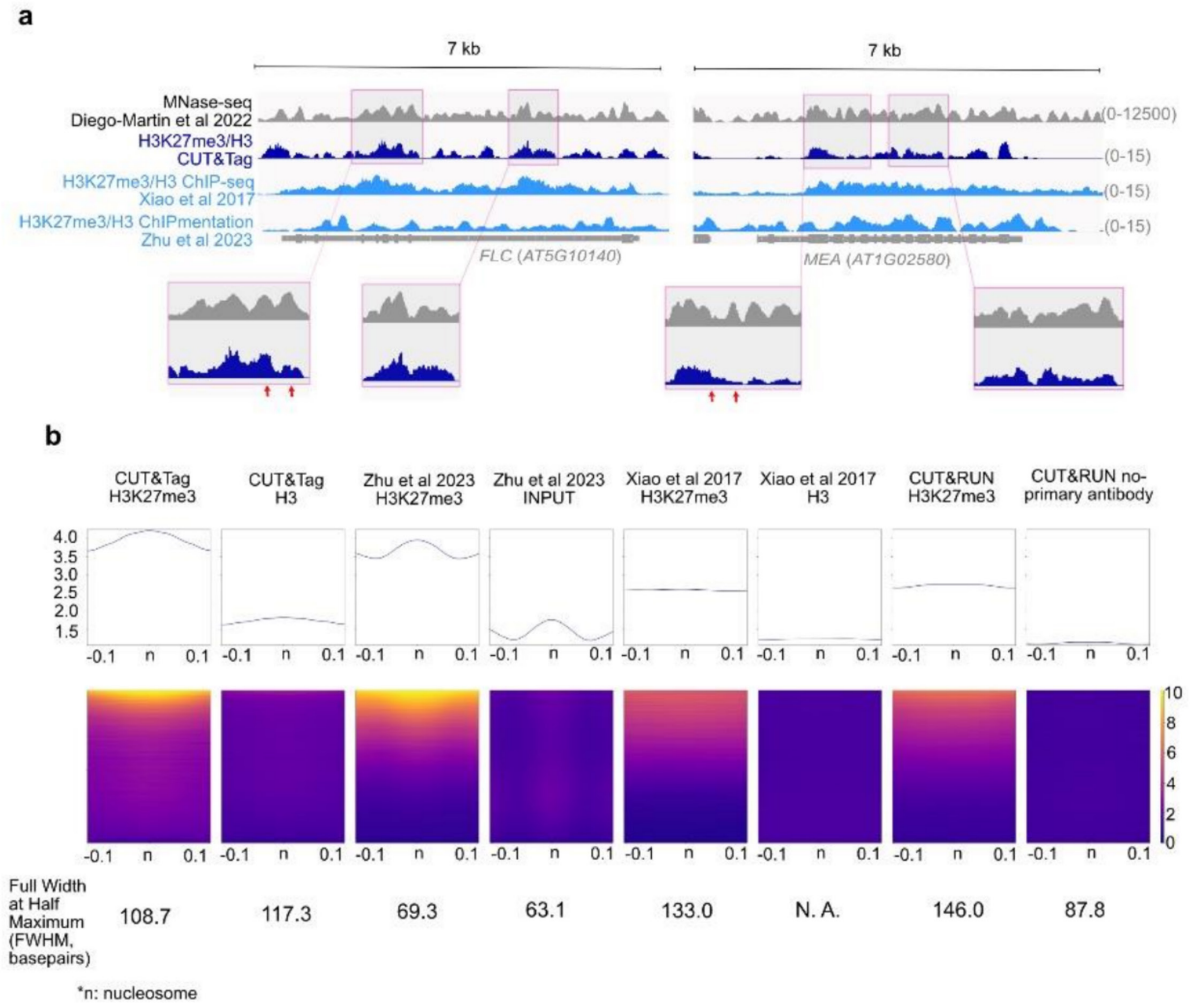
**CUT&Tag and ChIPmentation show resolutions close to nucleosome‐level.** (a) Genome browser view showing the comparison between the nucleosome occupancy (MNase‐seq) (Diego‐Martin et al. 2022) and H3K27me3 profiles from CUT&Tag and two ChIP‐seq datasets; regions in the CUT&Tag dataset show good correlation with the nucleosome occupancy are highlighted by a gray box; adjacent nucleosomes with strongly different H3K27me3 levels are pointed by red arrows. (b) Heatmap showing the distribution of H3K27me3 signal, and the control signals profiled by CUT&Tag, ChIPmentation, ChIP‐seq, and CUT&RUN around the H3K27me3‐related nucleosome centers (nucleosome centers that overlap with H3K27me3 peaks from at least one method). The full width at half maximum (FWHM, unit: base pairs) is indicated for the accumulated signal for each sample. N. A., not applicable, because the signal cannot be fitted with a sine function.

Histone modification profiles generated by CUT&Tag displayed a distinct wavy pattern, with signal peaks approximately 200–300 bp in width (Figure 6a), corresponding to the length of DNA wrapped around a nucleosome. This raised the question of whether CUT&Tag is capturing histone modifications at the level of individual nucleosomes, suggesting that it may offer nucleosome‐level resolution. To investigate this possibility, we compared the CUT&Tag data with a public MNase‐seq data of *Arabidopsis* seedlings (Diego‐Martin et al. 2022). We examined the H3K27me3 signal around two genes, which are H3K27me3‐marked in seedlings, *FLC* and *MEDEA* (*MEA*), and observed indeed that the CUT&Tag‐detected H3K27me3 signal and MNase‐seq showed overlapping wavy pattern in many regions (Figure 6a). Interestingly, we noticed that adjacent nucleosomes sometimes displayed markedly different levels of H3K27me3 enrichment (Figure 6a, red arrows). To determine whether the overlap between CUT&Tag and MNase‐seq data is a general feature at the genomic level, we used the nucleosome centers identified from MNase‐seq data to overlap with H3K27me3 peaks detected by at least one method. This allowed us to define a set of H3K27me3‐associated nucleosome centers. We then plotted heatmaps of CUT&Tag H3K27me3 and H3 signals centered on these positions (Figure 6b, first two columns at the left). Both signals showed clear enrichment around the nucleosome center, with a full width at half maximum (FWHM)—a measure of peak sharpness and resolution—of 108.7 bp for H3K27me3 and 117.3 bp for H3. These data support the conclusion that CUT&Tag can resolve histone modifications at single‐nucleosome resolution.

Next, we asked whether other profiling methods exhibit a similar ability to resolve nucleosome‐level patterns. Based on genomic tracks, ChIP‐seq (Xiao et al. 2017) appears to produce flatter, more averaged signals, whereas ChIPmentation (Zhu et al. 2023) displays a wavy pattern, though it differs from that seen in CUT&Tag (Figure 6a).

To compare across techniques, we generated heatmaps centered on the H3K27me3‐associated nucleosome positions for ChIP‐seq, ChIPmentation, and CUT&RUN datasets, along with their respective controls (Figure 6b). Among these, ChIPmentation showed the strongest enrichment at nucleosome centers, with FWHM values of 69.3 bp for H3K27me3 and 63.1 bp for the INPUT control. However, it is important to note that ChIPmentation involves MNase digestion to release nucleosomes (Zhu et al. 2023), which may contribute to the strong similarity to MNase‐seq profiles, as both rely on the same enzyme. In contrast, ChIP‐seq data showed no clear signal enrichment around nucleosome centers; the profiles were largely flat. CUT&RUN H3K27me3 data revealed only modest enrichment, while the control sample (no primary antibody) displayed weak but detectable signals.

In summary, CUT&Tag and ChIPmentation both produce wavy signal profiles consistent with nucleosome‐level resolution, where the patterns closely resemble those of MNase‐seq. This suggests that these two methods are capable of capturing histone modifications at the level of individual nucleosomes. By comparison, CUT&RUN shows lower resolution, and conventional ChIP‐seq lacks a detectable nucleosome‐level pattern.

## 3 Discussion

Here, we present an optimized and validated protocol for CUT&Tag targeting three different histone modifications in the tissues of the model plant *Arabidopsis thaliana* . This protocol primarily follows the steps outlined in previously published CUT&Tag protocols for mammalian cells (Kaya‐Okur et al. 2019; Kaya‐Okur et al. 2020), and we present an optimized variant that works efficiently with plant nuclei and a series of tested antibodies. Our results indicate that some antibodies, which perform well in ChIP‐seq may not work as well in CUT&Tag (Supplementary Figure S1). Therefore, we recommend using antibodies specifically tested for CUT&Tag when replicating these experiments.

Most of the datasets we generated are of high quality. However, it is important to note that the H3K27Ac and H3K4me3 datasets have relatively low numbers of reads mapping to the *Arabidopsis* genome (less than 2 million reads for some samples). This reduction is likely due to the use of a home‐made pA‐Tn5 enzyme that has been stored for an extended period, leading to elevated levels of tagmented *E. coli* DNA (see Methods section and Supplementary Figure S8). Despite the low number of *Arabidopsis*‐aligned reads, replicates for both H3K27Ac and H3K4me3 exhibited good quality metrics, including robust peak calling performance and acceptable FRiP (fraction of reads in peaks) scores (Supplementary Figure S1.2). The third replicate for each histone modification showed reduced peak‐calling performance and was therefore not used for quantitative analysis. However, because these replicates clustered well with the others (Supplementary Figure S4), they were retained for genome browser visualization.

By comparing our CUT&Tag‐generated datasets with two publicly available ChIP‐seq datasets, we showed that CUT&Tag is a valid method for epigenome profiling and explored the differences and the advantages of CUT&Tag versus ChIP‐seq. Indeed, CUT&Tag captures the genome‐wide distribution of three histone modifications: H3K27me3, H3K4me3, and H3K27Ac from a small amount of material and also reaches a resolution allowing visualization of the epigenetic states on individual nucleosomes, similar to ChIPmentation, but higher compared to conventional ChIP‐seq.

ChIP‐seq and CUT&Tag are orthogonal methods for epigenome profiling, and there have been some reports stating that they reveal some different sets of peaks (Kaya‐Okur et al. 2019; Tao et al. 2020; Ouyang et al. 2021; Abbasova et al. 2025). In our work, we also found that CUT&Tag and ChIP‐seq both captured common but also sets of peaks specific to themselves. The ChIP‐seq specific peaks are enriched in intergenic regions with low read density. Considering that ChIP‐seq is known to produce low‐density genome‐wide noise (Skene and Henikoff 2017), many of the intergenic ChIP‐specific peaks could possibly be noise from low‐signal regions.

We also found that the CUT&Tag‐specific peaks are enriched on exons, suggesting CUT&Tag has an increased sensitivity in detecting histone modifications in exon‐rich regions. It is important to notice that this enrichment may be a result of differences in GC content, as exons generally have higher GC content than introns. Indeed, when comparing exons and introns with matched GC content, H3K27me3 signal levels were similar.

However, the observed exon bias is unlikely to be caused by mapping artifacts, as over 95% of fragments from the CUT&Tag H3K27me3 libraries successfully mapped to the *Arabidopsis* genome. We therefore hypothesize that the apparent enrichment in exons may reflect a biological feature—namely, that nucleosomes and H3K27me3 are more frequently associated with GC‐rich regions, which often correspond to exonic sequences.

While GC content may explain the general trend, it remains intriguing that exon enrichment is more pronounced in CUT&Tag and ChIPmentation datasets than in conventional ChIP‐seq. We propose that this difference could be due to the higher resolution and reduced background of CUT&Tag and ChIPmentation, which allows these techniques to more accurately resolve histone modification patterns at the level of individual nucleosomes.

The average lengths of exons and introns of *Arabidopsis* are around 250 and 168 bp, respectively (Initiativegenomeanalysis@tgr.org genomeanalysis@gsf.de TAG 2000; Chang et al. 2017). Because of the lower resolution of conventional ChIP‐seq, it is difficult to accurately distinguish histone modification patterns on introns and exons. This could explain why exon‐specific enrichment is less apparent in ChIP‐seq data. An alternative explanation is that the observed exon enrichment in CUT&Tag and ChIPmentation may reflect the sequence bias of the Tn5 transposase, which is used in both protocols for library preparation. However, the pronounced differences in H3K27me3 signal between exons and introns—visible in many individual genes (e.g., Figure 3b)—suggest that Tn5 bias alone cannot account for the observed pattern. In addition, as shown in the heatmap (Figure 3c), a modest degree of exon enrichment is also observed in CUT&RUN and ChIP‐seq datasets, where no Tn5 transposon is used. This supports the idea that exon enrichment of H3K27me3 is a genuine feature detectable across multiple profiling methods, and is unlikely to be solely the result of enzyme sequence preference.

It has been known that nucleosomes and some histone modifications are more enriched in exons than introns, which may instruct splicing (Agirre et al. 2021; Kolasinska‐Zwierz et al. 2009). Regardless of GC‐content or not, the enrichment of H3K27me3 on *Arabidopsis* gene exons has not been previously reported. Given H3K27me3's significant role in gene repression and development, investigating its impact on splicing and its interaction with known functions of H3K27me3 would be valuable. Also, the degree of exon enrichment of H3K27me3 seems to be stronger in *Arabidopsis* compared to that in animal cells (Andersson et al. 2009), possibly suggesting an interesting crosstalk between splicing and chromatin structure in *Arabidopsis*, which could be an interesting avenue of research.

We observed weak enrichment of the histone modification H3K27me3 on the gene bodies of many genes, including housekeeping genes, which are actively expressed in seedlings and are not expected to be marked by H3K27me3. We speculate that this observation could be due to the increased sensitivity of CUT&Tag, which allows for the detection of weak signatures of H3K27me3 enrichment on exons. Moreover, given that our starting material—the *Arabidopsis* seedling—consists of a mixture of different cell types and that epigenetic states are cell type‐specific, this weak enrichment might come from H3K27me3 occupancy in a small subset of cells. Therefore, although CUT&Tag performed on bulk tissues like seedlings does not directly reveal the epigenetic states of individual cell types, we propose that combining this technique with gene expression data from individual lineages could provide insights into the epigenetic landscape across different cell types. More tailored studies are needed to correlate gene expression patterns with observed histone mark enrichment, thereby inferring the epigenetic states within distinct cell populations.

We showed that an advantage of CUT&Tag versus ChIP‐seq is its compatibility with small amounts of starting material. In this work, we used just a few 1‐ or 2‐week‐old seedlings for each reaction, which was about 0.01 g instead of gram‐level amounts required by ChIP‐seq. By this, CUT&Tag can enable research with limits in material availability, to name some examples, the research of epigenetic variation between individual plants or the research of epigenetic states of certain tissue types during flower development.

By comparing the data to the nucleosome distribution from MNase‐seq data, we demonstrated that CUT&Tag achieves a resolution close to the nucleosome level, thus providing an accurate depiction of the epigenetic status of a gene. We showed that this signature is similar to that of ChIPmentation, where single nucleosomes are released from chromatin by MNase. While in ChIP‐seq, the nucleosome patterns are often ambiguous, and histone modifications are generally detected spread across long genomic regions. By visually inspecting the CUT&Tag data, we showed that even adjacent nucleosomes within H3K27me3‐enriched regions can exhibit markedly different levels of H3K27me3. This variation in epigenetic mark levels at the nucleosome level presents an intriguing question for further investigations, and CUT&Tag would be a valuable technique for such investigations.

## 4 Conclusions

We presented a CUT&Tag protocol for the model plant Arabidopsis. CUT&Tag captures the genome‐wide distribution of histone modifications while using much less material than ChIP‐seq. Additionally, the CUT&Tag data has a higher resolution compared to conventional ChIP‐seq.

## 5 Methods

### 5.1 Plant Material and Growth Conditions

*Arabidopsis thaliana* ecotype Col‐0 (Columbia) NASC #N70000 seeds were sown on half‐strength MS media (1/2 MS salt base [Carolina Biologicals, USA], 1% sucrose, 0.05% MES, 0.8% Phytoagar [Duchefa], pH > 5.7 with KOH) and subjected to stratification at 4°C for 2–3 days. Following this, the plates were moved to a plant incubator (CU36L6, CLF Plant Climatics) set to long‐day conditions (8 h dark at 18°C, 16 h light at 22°C, 70% relative humidity).

When harvesting seedlings, the plants were grown on half‐strength MS media for between 1 and 2 weeks, allowing for the development of four to eight true leaves.

### 5.2 Antibodies Used in This Study

Rabbit anti‐H3K27me3: Cell signaling technology #9733.

Rabbit anti H3K4me3: Diagenode c15410003.

Rabbit anti H3K27Ac: Abcam #4729.

Mouse anti‐H3: Active Motif 39064.

Guinea Pig anti‐Rabbit IgG: antibodies‐online #ABIN101961.

Rabbit anti‐Mouse IgG: Abcam 46540.

### 5.3 Production of pA‐Tn5

The procedure for purifying pA‐Tn5 is based on previously reported protocols (Kaya‐Okur et al. 2019; Li et al. 2021) and the workflow is summarized in Supplementary Figure S9a.

HEGX buffer and dialysis buffer were prepared before starting the protein production. HEGX buffer is prepared using the following recipe: 20 mM HEPES pH 7.2 (Sigma‐Aldrich #H3375), 1 M NaCl (Roth #3957.1), 1 mM EDTA pH 8 (HUBERLAB # A2937.1000), 10% Glycerol (Roth #3783.1), 0.2% Triton X‐100 (Sigma #T8787), Protease Inhibitor (1×, add freshly before use) (Roche #64178100). Dialysis buffer is prepared using the following recipe: 100 mM HEPES pH 7.2 (Sigma‐Aldrich #H3375), 200 mM NaCl (Roth #3957.1), 0.2 mM EDTA (HUBERLAB # A2937.1000), 20% Glycerol (Roth #3783.1), 0.2% Triton X‐100 (Sigma #T8787), and 2 mM DTT (Merck # 1.24511.0005).

*E. coli* containing the plasmid the 3XFlag‐pA‐Tn5‐Fl (Addgene Plasmid #124601, C3013 *E. coli* strain) was obtained from Addgene, and propagated on a plate by streaking the bacteria from the agar stab onto an LB agar plate supplemented with 100 μg/mL Ampicillin (Applichem #A0839) and let to grow overnight at 37°C. A single colony was then inoculated in 7.5 mL of liquid LB medium supplemented with 100 μg/mL Ampicillin and allowed to grow at 37°C, shaking at 180 rpm for about 4 h. This 7.5 mL of culture was then added to 500 mL of liquid LB medium supplemented with 100 μg/mL Ampicillin and left to grow at 37°C, shaking 180 rpm until an OD600 value between 0.5 and 0.7 was reached. Then, the liquid culture was cooled down on ice for 1 h, and 125 μL of 1 M IPTG (final concentration 0.25 mM) was added to induce the expression of the protein, overnight at 18°C shaking at 180 rpm. The following morning, the culture was centrifuged at 9500 × g at 4°C for 1 h to pellet the bacteria using a Sorvall LYNX 6000 Superspeed Centrifuge (Thermo Scientific #75006590), the supernatant removed, and the bacteria pellet frozen at −80°C for at least 2 h. To purify the protein, the bacteria pellet was thawed on ice, resuspended in five volumes of HEGX buffer supplemented with 0.5 mg/mL Lysozyme (Roche #10837059001), and incubated on ice for 30 min. After the incubation, the bacteria suspension was sonicated with a Bioruptor bath sonicator (Diagenode), with setting M, 15 s on, 15 s off, 10 cycles. The lysate was then centrifuged at 31,000 × g, 4°C, for 30 min and the supernatant mixed with 5 mL Chitin Resin (NEB #S6651), previously washed first with 30 mL of ddH_2_O and then 20 mL of HEGX buffer. The samples were incubated at 4°C for 48 h while rotating. After incubation, the sample was transferred into a 10 mL gravity column (Thermo Scientific #89898). Once all the resin was packed in the gravity column, the resin was washed with 20 mL HEGX buffer supplemented with protease inhibitor (1×, Roche #64178100), and then the protein was eluted with elution buffer (HEGX buffer with 100 mM DTT [Merck # 1.24511.0005]). The protein prep was then dialyzed with SnakeSkin Dialysis Tubing (3500 MWCO, Thermo Scientific #68035) using 800 mL of dialysis buffer at 4°C. The quality of the purified pA‐Tn5 protein was confirmed by SDS‐PAGE followed by Coomassie blue staining and its concentration was estimated by comparing to BSA standards on the same gel. Then, the protein was concentrated by centrifugation with a Pierce Protein Concentrator (10 K MWCO, Thermo Scientific #88516) to a concentration close to 800 ng/μL. An equal volume of filter‐sterilized 80% Glycerol (Roth #3783.1) was added to the protein prep, bringing the protein concentration to about 400 μg/mL (about 5.5 μM). The protein was aliquoted, and aliquots were stored at −20°C.

DNA oligos used to create the adaptors, including ME‐A (sequence: TCGTCGGCAGCGTCAGATGTGTATAAGAGACAG), ME‐B (sequence: GTCTCGTGGGCTCGGAGATGTGTATAAGAGACAG), and MErev (sequence: /5Phos/CTGTCTCTTATACACATCT) were synthesized with standard desalt purification and resuspended in annealing buffer (10 mM Tris–HCl pH 8.0, 50 mM NaCl, 1 mM EDTA) to create a stock concentration of 200 mM. To form ME‐A and ME‐B adaptors, the pair ME‐A plus MErev (40 μL each) and ME‐B plus MErev (40 μL each) were mixed, heated at 95°C for 5 min, and left to cool to room temperature in the heating block turned off. The formed ME‐A and ME‐B adaptors were then stored at 4°C. To load pA‐Tn5 with the adaptors, 4 μL ME‐A adaptor, 4 μL ME‐B adaptor, and 50 μL pA‐Tn5 (at approximately 400 μg/mL) were mixed and incubated at room temperature for 50 min and then stored at −20°C.

To test the transposase activity of the loaded pA‐Tn5, 1.25 μL of loaded pA‐Tn5 was added to 1 μL of a plasmid at 250 ng/μL (we used a derivative of pAGM65879 [Addgene #153214]), 1 μL NEBuffer 3.1 (NEB #B6003S), 6.75 μL ddH_2_O, and incubated at 37°C for 15 min. To stop the reaction, 1 μL of 1% SDS (Roth #CN30.3) was added, the sample incubated at 55°C for 10 min, and then 0.32 μL Proteinase K (Thermo Scientific #EO0492) was added, and the sample incubated further at 55°C for 10 min. Complete digestion of the plasmid, as expected if pA‐Tn5 has a good transposase activity, was evaluated by loading the sample on 1% Agarose gel (Supplementary Figure S9b,c).

The loaded and unloaded pA‐Tn5 are stored at −20°C. Please note that the purified pA‐Tn5 stock contains a trace amount of *E. coli* DNA, and the loaded pA‐Tn5 can slowly tagment the *E. coli* DNA even if stored at −20°C, leading to an increased tagmented *E. coli* DNA level and a decrease in activity (Supplementary Figure S10) (Kaya‐Okur et al. 2019). Therefore, the tagmented pA‐Tn5 should not be stored for more than six months.

### 5.4 Reagent Setup for Nuclei Extraction

#### 5.4.1 LB01 Buffer for Nuclei Extraction (40 mL)

0.6 mL 1 M Tris–HCl, pH 8 (Roth # AE15.3) (final: 15 mM).

0.16 mL 0.5 M EDTA (HUBERLAB #A2937.1000) (final: 2 mM).

20 μL 1 M Spermidine (Spermidine trihydrochloride: Sigma‐Aldrich #S2876) (final: 0.5 mM).

1.6 mL 2 M KCl (Roth #6781.1) (final: 80 mM).

0.16 mL 5 M NaCl (Roth #3957.1) (final: 20 mM).

0.8 mL 5% Triton (Sigma #T8787) (final: 0.1%).

36.66 mL ddH_2_O.

Store at 4°C for short‐term uses. Make 10 mL aliquots and freeze at −20°C for long‐term storage. The 1 mL LB01 buffer is used for nuclei extraction from each seedling sample contained in one 1.5‐mL tube.

Add 1/25 volume of Protease Inhibitor (cOmplete, EDTA‐free Protease Inhibitor Cocktail, Roche #6417810). (25×) before use.

#### 5.4.2 Honda Buffer for Nuclei Extraction (40 mL)

1.6 mL 0.5 M HEPES, pH 7.5 (Sigma‐Aldrich #H3375) (final: 20 mM).

17.6 mL 1 M Sucrose (PanReac AppliChem #A2211) (Final: 0.44 M).

6.2 mL 8% Ficoll PM400 (Merck #F437) (Final: 1.25%).

8 mL 12.5% Dextran T‐40 (Roth #7626.4) (final: 2.5%).

0.4 mL 1 M MgCl_2_ (MgCl_2_*6H_2_O Sigma‐Aldrich # M2670) (10 mM).

4 mL 5% TritonX‐100 (Sigma #T8787) (final 0.5%).

0.2 mL 1 M DTT (Merck # 1.24511.0005) (Final: 5 mM).

0.4 mL ddH_2_O.

Store at 4°C for short‐term uses. Make 5 mL aliquots and freeze at −20°C for long‐term storage. 1 mL LB01 buffer is used for nuclei extraction from each seedling sample contained in one 1.5‐mL tube.

1/25 volume of Protease Inhibitor (cOmplete, EDTA‐free Protease Inhibitor Cocktail, Roche #6417810) (25×) before use.

### 5.5 Nuclei Extraction From Seedlings

The aerial parts of two to four seedlings between one and 2 weeks old were collected to extract the nuclei for each CUT&Tag reaction. The seedlings were collected into a 1.5‐mL DNA LoBind tube (Eppendorf #L203809K). The tubes containing the sample were snap frozen in liquid nitrogen, and then ground with a Silamat S6 tissue grinder (or similar) for 5 s while the tissue was still frozen. The freeze‐grind cycle was repeated for a total of three times for each tube. After grinding, 800 μL of Honda buffer was added to each tube, and the sample was then incubated on ice for 20 min. The sample was then filtered through a 30‐μm CellStrainer (CellTrics) into a clean 2‐mL centrifuge tube. An additional 600‐μL Honda buffer was used to wash the 1.5‐mL tube and the Cell Strainer and merged to the initial 800 μL in the appropriate 2‐mL sample tube. A final volume of approximately 1.4 mL was obtained at this step. The nuclei samples were then centrifuged at 4000 × g for 15 min at 4°C, and then the supernatant was removed. To wash the nuclei pellet, 1 mL LB01 buffer was added. The tubes were then centrifuged again at 1000 × g at 4°C for 15 min, and the supernatant was removed. The nuclei pellet was then resuspended in 200 μL of Wash Buffer. This step uses an EDTA‐containing buffer (LB01) to remove the residual Mg^2+^ from the Honda buffer, and then the nuclei are resuspended in an EDTA‐free buffer (Wash Buffer). The quality of the nuclei was checked using a microscope before proceeding with the CUT&Tag.

### 5.6 Nuclei Quality Check

For quality control, 3 μL of the nuclei suspension was mixed with 3 μL of VECTASHIELD Antifade mounting medium supplemented with DAPI (Vector Laboratories, H‐1200). The sample was loaded on a Neubauer hemocytometer (VWR #631‐0926), and the nuclei's integrity and number were assessed with a Leica DM6000 fluorescence microscope. The nuclei of Arabidopsis have diameters between 1 and 10 μm, generally with a smooth contour. A nucleus can be recognized by its chromocenters and sometimes the nucleolus. A good nucleus may have 1–10 chromocenters.

### 5.7 Reagent Setup for CUT&Tag

#### 5.7.1 Binding Buffer for Activating Con‐A Beads (10 mL)

400 μL 0.5 M HEPES (Sigma‐Aldrich #H3375), pH 7.9.

50 μL 2 M KCl (Roth #6781.1).

10 μL 1 M CaCl_2_ (Sigma‐Aldrich #21074).

10 μL 1 M MnCl_2_ (Merck # 1.05927.0100).

9.5 mL ddH_2_O.

Store at 4°C for up to 6 months.

#### 5.7.2 Wash Buffer (50 mL)

2 mL 0.5 M HEPES (Sigma‐Aldrich #H3375), pH 7.5 (final: 20 mM).

1.5 mL 5 M NaCl (Roth #3957.1) (Final: 150 mM).

25 μL 1 M Spermindine (Spermidine trihydrochloride: Sigma‐Aldrich #S2876) (final: 0.5 mM).

2 mL 25× Protease Inhibitor Cocktail (cOmplete, EDTA‐free Protease Inhibitor Cocktail, Roche #6417810) (final: 1×).

Add ddH2O to a final volume of 50 mL, filter sterilize.

#### 5.7.3 Tween‐Wash Buffer (20 mL)

550 μL needed for each reaction; prepared by adding 400 μL 5% Tween 20 (Sigma #P9416) (final: 0.1%) to 20 mL Wash Buffer.

#### 5.7.4 Antibody Buffer (1 mL)

50 μL needed for each reaction; prepared by adding 4 μL 0.5 M EDTA (HUBERLAB #A2937.1000) (final 2 mM) and 10 μL 10% BSA (Sigma‐Aldrich #A4503) (final 0.1%).

1 mL Tween‐Wash Buffer.

#### 5.7.5 300‐Wash Buffer (50 mL)

2 mL 0.5 M HEPES (Sigma‐Aldrich #H3375), pH 7.5.

3 mL 5 M NaCl (Roth #3957.1) final 300 mM.

25 μL 1 M Spermindine (Spermidine trihydrochloride: Sigma‐Aldrich #S2876).

2 mL 25× PI (cOmplete, EDTA‐free Protease Inhibitor Cocktail, Roche #6417810) (final: 1×).

ddH2O to a final volume of 50 mL, filter sterilize.

*300 stands for the concentration of NaCl in the buffer.

#### 5.7.6 Tween‐300‐Wash Buffer (20 mL)

760 μL needed for each reaction; prepared by adding 40 μL 5% Tween 20 (Sigma #P9416) (final: 0.01%) to 20 mL of 300‐Wash Buffer.

#### 5.7.7 Tagmentation Buffer (4 mL)

300 μL needed for each reaction; 4 mL Tween‐300‐wash buffer + 40 μL 1 M MgCl2 (MgCl_2_*6H_2_O Sigma‐Aldrich # M2670) (final: 10 mM).

### 5.8 CUT&Tag

#### 5.8.1 (Day 1)

##### 5.8.1.1 Preparing Concavalin‐A (Con‐A) Beads

1 Resuspend Concavalin‐A beads (BioMag Plus Concanavalin A, Polyscience Inc., #86057‐10) by shaking the bottle. Transfer enough volume to a 2‐mL tube containing 1.6 mL of Binding Buffer and incubate for 2 min. 15 μL of Con‐A beads were used for each sample.
2 Put the tube on a magnet stand (Invitrogen #CS15000) and wait until the supernatant becomes clear (takes 1–2 min). Then, remove the supernatant using a 1000 uL pipette.
3 Resuspend the beads using another 1.5 mL Binding Buffer by pipetting up and down. Then, put the tube on the magnet stand, wait until clear, and remove the supernatant.
4 Pipette to resuspend the Con‐A beads with Binding buffer; the volume of the binding buffer to use here is the same as the volume of Con‐A beads used in step 1. The Con‐A beads are now activated.

##### 5.8.1.2 Binding of Nuclei to Con‐A Beads and Binding of Primary Antibody

5 Dispense 15 μL of the activated Con‐A beads to each sample of nuclei (which are in the Wash Buffer). Rotate the mixture of the nuclei and beads at room temperature for 10 min.
6 Meanwhile, dilute the primary antibody of choice in the antibody buffer. For each sample, prepare 50 μL of antibody buffer, and add antibody at a dilution ratio of 1:50, or follow the dilution ratio suggested by the antibody provider or previous reports.
7 After the 10 min of incubation (step 5) have passed, put the tubes on the magnet stand, wait until the supernatant is clear, then remove the supernatant with a P200 pipette. Immediately resuspend each sample in the 50 μL antibody buffer prepared in step 6.
8 Put the samples to rotate at 4°C and incubate overnight.

#### 5.8.2 (Day 2)

##### 5.8.2.1 Binding of Secondary Antibody

9 Dilute the secondary antibody of choice in Tween‐Wash Buffer (55 μL for each sample)
10 After the overnight incubation of the sample, quickly spin the tubes with a microcentrifuge to collect all the liquid on the bottom of the tubes, then place the tubes on the magnet stand.
11 Using a P200 pipette set at 55 μL, remove the supernatant, and then immediately add 55 μL of the dilution of the secondary antibodies.
12 Resuspend the beads by gently vortexing the tube and incubate while rotating at room temperature for 45 min.

##### 5.8.2.2 Binding of the pA‐Tn5

13 Dilute the pA‐Tn5 (Loaded with adaptors, 4.74 uM) at 1:250 in Tween‐300‐Wash Buffer.
14 After the 45‐min incubation (step 3.4), briefly spin the samples, put them on a magnet stand, and use a P200 pipette set at 60 μL to completely remove the supernatant. Then, while the tubes stay on the magnet stand, add 500 μL Tween‐wash buffer to each tube.
15 Remove the majority of the supernatant first with the P1000 pipette, then the remaining with the P200 pipette. If liquid is attached to the wall of the tube, briefly spin the tube to collect the liquid at the bottom of the tube and then completely remove it with the P200 pipette.
16 Add 60 μL pA‐Tn5 dilution and resuspend by gently vortexing the tube.
17 Rotate at room temperature for 1 h.

##### 5.8.2.3 Tagmentation

18 After the incubation (step 17), briefly spin the samples and put them on the magnet stand, then remove the supernatant with a P200 pipette.
19 While keeping the tubes on the magnet stand, add 500 μL of Tween‐300‐Wash Buffer to each sample for a wash. Remove the tubes from the magnet stand, shake briefly to mix, put them back onto the magnet stand, and wait until clear.
20 Completely remove the liquid, and add 200 μL Tween‐300‐Wash Buffer without mixing for the second wash. Remove the tubes from the magnet stand, shake briefly to mix, put them back onto the magnet stand, and wait until clear.
21 Completely remove the liquid, add 300 μL Tagmentation Buffer to each tube, and mix well by gently vortexing.
22 Incubate the samples on a heating block at 37°C for 1 h.

##### 5.8.2.4 Release of DNA Fragments

23 After the incubation (step 22), add to each tube 10 μL 0.5 M EDTA (HUBERLAB # A2937.1000), 3 μL 10% SDS (Roth #CN30.3), and 2.5 μL 20 mg/mL Proteinase K (Thermo Scientific #EO0492) to stop the tagmentation.
24 Mix by vortexing, then incubate at 55°C on a heating block for 1 h.

##### 5.8.2.5 DNA Extraction With PCI

25 Pre‐cool some 100% ethanol (Fisher Chemical E/0665DF/17) and prepare enough 1.5‐mL tubes as the number of samples with 750 μL pre‐cooled 100% ethanol.
26 To each sample (from step 6.2), add 300 μL phenol:chloroform:isoamyl alcohol 25:24:1 (PCI) (Sigma‐Aldrich #77617), and vortex for 2 s.
27 Transfer the mixture to a phase‐lock tube, centrifuge for 3 min at 16,000 × g at room temperature.
28 Add 300 μL chloroform (scharlau #CL02181000) to each phase‐lock tube and invert to mix; centrifuge for 3 min at 16,000 × g at room temperature to remove the residual phenol in the aqueous phase.
29 Transfer the aqueous layer (the upper layer) to the tubes with 750 μL ethanol (prepared in step 25). Be careful not to stab the pipette tip into the gel of the phase‐lock tube.
30 Chill the tubes on ice for 5–10 min and centrifuge for 20 min at 16,000 × g at 4°C.
31 Gently pour out the supernatant and use a piece of paper towel to remove the liquid completely. The DNA pellet is attached to the wall of the tube, although usually not visible.
32 Air dry the tube for 5–10 min, then add 1 mL pre‐cooled 100% ethanol to wash.
33 Centrifuge at 16,000 × g for 10 min at 4°C.
34 Gently pour out the supernatant and use a piece of paper towel to completely remove the liquid. Then, air dry the tube until no liquid is visible: it takes about 20 to 30 min. Add 22 μL 0.1× TE buffer (1 mM Tris [Roth # AE15.3], pH 8, 0.1 mM EDTA [HUBERLAB # A2937.1000]) and vortex well to dissolve the DNA.

##### 5.8.2.6 PCR Amplification for Tagmented Fragments

35 In a PCR tube (Thermo Fisher Scientific #AB0451), mix 21 μL tagmented DNA, 2 μL i5 primer (10 μM) and 2 μL i7 primer (10 μM), and 25 μL NEBNext Hi‐Fi 2× PCR mix (NEB #M0541) to make a total final volume of 50 μL. Make sure to use unique combinations of i5 and i7 primers for each library that will be sequenced in the same pool. The sequences of i5 and i7 primers are from Kaya‐Okur et al. (2020) and can be found in Supplementary Table S3.
36 On a thermocycler, perform the PCR with the program listed in Table 2.

**TABLE 2:** PCR cycle program for CUT&Tag.

Temperature	Time	Ramp rate	Repeat
58°C	5 min		
72°C	5 min		
98°C	45 s		
98°C	15 s	↘ 3°C/s	*15×
63°C	10 s	↗ 3°C/s	
72°C	1 min		
12°C	∞		

*Note:* The cycle number may need optimization depending on the amount of starting material. Please be cautious with increasing the PCR cycle number, as this may lead to unwanted duplication levels that will reduce the quality of the results.

##### 5.8.2.7 PCR Purification

37 Once the PCR is completed (step 36), to each sample add 1.1 volume (55 μL) of SPRI beads (NucleoMag # 744970) to each PCR reaction, pipette up and down 10 times to mix well, and leave at room temperature for 10 min.
*Note:* purification using 1.1 volume of SPRI beads would reduce the amount of short fragments between 200–300 bp; alternatively, one can use 1.3 volume to preserve more fragments, but the short fragments between 200 and 300 will be dominant in this case
38 Place the PCR tubes on a magnet stand (Invitrogen), wait until the supernatant is clear, then remove the liquid with a 200 μL pipette set at 180 μL.
39 While the tubes stay on the magnet stand, add 180 μL 80% ethanol (Fisher Chemical E/0665DF/17) to wash the beads.
40 Remove the liquid with the 200 μL pipette set at 190 μL, then add another 190 μL of 80% ethanol for the second wash.
41 Remove the liquid with the 200 μL pipette set at 200 μL. To remove the liquid attached to the tube wall, briefly spin the tubes, then put them back on the magnet stand, and remove the liquid with the pipette completely.
42 Briefly air dry the beads for less than 5 min, then add 25 μL ddH_2_O to elute the DNA. Vortex and wait for 5 min at room temperature.
43 Put the sample back on the magnetic stand, wait until the supernatant becomes clear, then carefully transfer the liquid to a PCR tube (Sarstedt #72.991.002).

##### 5.8.2.8 Library Profile and Quantitation

44 Use 2 μL of each library to visualize the size distribution with a High Sensitivity D1000 (HSD1000) tape (Agilent 5067‐5584) on an Agilent Tapestation 4200.

*Note:* Do not use agarose as the libraries are usually not visible on agarose gels.

45 Select a region from 170 to 625 bp to quantify the concentration, as this is the range of size that will be mostly captured by the Illumina sequencers.
46 Proceed to library pooling and next‐generation sequencing, or store the libraries at −20°C.

##### 5.8.2.9 Next‐Generation Sequencing

47 Pool the libraries to the desired concentration, targeting to reach an equal molarity for each library.
48 Sequence the libraries with a short‐read sequencer. The choice of sequencer and flowcell depends on the target read numbers. Aim for 10–30 million reads for each H3K27me3, H3K4me3, or H3K27Ac library.

### 5.9 Analysis of CUT&Tag Data

Data analysis of CUT&Tag has been performed based on the tutorial from Zheng et al. (2020) (Henikoff et al. 2020; Zheng et al. n.d.). Reads were quality‐checked using fastqc (0.12.1) and trimmed with fastp (Version 0.23.4, ‐l 30 ‐‐trim_poly_x ‐‐poly_x_min_len 32) (Chen et al. 2018). Trimmed reads were aligned to the *A. thaliana* reference genome (TAIR10) using bowtie2 (Version 2.4.1, options ‐‐local ‐‐very‐sensitive ‐‐no‐mixed ‐‐no‐discordant ‐‐phred33 ‐I 10 ‐X 700) (Langmead and Salzberg 2012). Duplicate reads were marked with Picard tools (version 3.1.1, broadinstitute.github.io/picard). Fragment count in 500 bp genomic bins are extracted using samtools (Version 1.17) (Danecek et al. 2021) and the awk command following the code from Zheng et al. (2020) (awk ‐F'\t' 'function abs(x) {return ((x < 0.0)? ‐x: x)} {print abs($9)}'|sort|uniq ‐c|awk ‐v OFS = "\t" '{print $2, $1/2}') (Henikoff et al. 2020; Zheng et al. n.d.) and the correlation indexes were calculated in R (Version 4.3.1). Peak calling was done using MACS2 (Version 2.2.9.1, options ‐f BAMPE ‐q 0.05 ‐‐broad ‐‐broad‐cutoff 0.1 ‐B ‐‐SPMR) (Zhang et al. 2008). Peaks reproduced in two out of three replicates are considered consensus peaks (Yang et al. 2014). These consensus peaks are extracted using bedtools intersect (Version 2.31.0) (Quinlan and Hall 2010) and are used for the comparisons. To calculate the fraction of reads in peaks (FRiP) score, the .bam files after duplication removal were transformed to .bed files, and the number of reads in peaks was obtained by intersecting the .bed files with peak files using bedtools (Version 2.31.0) (Quinlan and Hall 2010). The distribution of genomic features on peaks was plotted using ChIPseeker (Version 1.38.0) (Yu et al. 2015).

For visualization on the genome browser, the coverage normalized by sequencing depth was calculated by bamCoverage from deepTools (Version 3.5.3, ‐‐binSize 10 ‐‐normalizeUsing RPGC ‐‐effectiveGenomeSize 119481543 ‐‐ignoreForNormalization chrC, chrM ‐‐extendReads) (Ramírez et al. 2016) and visualized with the Integrative Genomics Viewer (IGV) (Thorvaldsdóttir et al. 2013). To visualize the histone modification with background correction by the H3 controls, .bedGraph files for the histone modifications and control lambda were generated by MACS2 during peak calling with the “‐B ‐‐SPMR” flag. Background corrected .bedGraph files were generated using the bdgcmp function of MACS2 and converted to .bw files using bedGraphToBigWig (Version 445) (Kent et al. 2010) with the FE (Fold Enrichment) option.

To associate peaks with genes, peaks are annotated using ChIPseeker (Version 1.38.0) (Thorvaldsdóttir et al. 2013); then, peaks with the “distal intergenic” were excluded from further analysis. The overlaps of marked genes were plotted using VennDiagram (Version 1.7.3) (Chen and Boutros 2011) in R (v4.3.1).

### 5.10 Comparison of CUT&Tag With ChIP‐seq, ChIPmentation, CUT&RUN, and MNase Datasets

To compare our results with previously published ChIP‐seq datasets, we downloaded the raw sequencing data from NCBI GEO (GSE84483, Xiao et al. 2017, ChIP‐seq data) and ArrayExpress (E‐MTAB‐10966, Zhu et al. 2023, ChIPmentation data). We processed these public ChIP‐seq and ChIPmentation datasets using the same pipeline applied to our CUT&Tag data, with modifications to accommodate single‐end sequencing data for GSE84483.

The Venn plots showing the overlap between peaks and the distribution of genomic features on peaks were plotted using ChIPpeakAnno (makeVennDiagram and genomicElementDistribution functions, Version 3.36.1) (Zhu et al. 2010). Numbers of reads on peaks were counted using featureCounts (Version 2.0.6) (Liao et al. 2014). The comparison of peak lengths and read counts in peaks was plotted in R (v4.3.1).

The CUT&RUN raw data were downloaded from NCBI GEO (GSE123602) and were processed following the same pipeline applied to our CUT&Tag data. The processed .bigwig files of the MNase‐seq data were downloaded from NCBI GEO (GSE205110) (Diego‐Martin et al. 2022).

To generate the heatmaps, the sequence‐depth‐normalized bigwig files were generated from bam files using deepTools bamcoverage (Version 3.5.3) (Ramírez et al. 2016) with options ‘‐binSize 10 ‐‐normalizeUsing RPGC ‐‐effectiveGenomeSize 119481543 ‐‐ignoreForNormalization chrC, chrM –extendReads (no parameter for double‐end data; 200 for single‐end data).’ Duplicate‐removed bam files from the first replicate of each method are used, except for the ChIPmentation H3K27me3 data, where two replicates were merged to give similar total read counts compared to those of CUT&Tag and ChIP‐seq H3K27me3 data.

Exon bed files are extracted from the gene annotation GFF3 file downloaded from TAIR10 (TAIR10_GFF3_genes.gff). The first and the last exons are excluded; therefore, all the exons used are flanked by introns. Then, the exon files are overlapped with H3K27me3 peaks called from all methods, to keep these exons overlapped with H3K27me3 peaks from at least one method. The bed file for nucleosome is acquired by contacting the authors of Diego‐Martin et al. (2022). Then, the nucleosome bed file is also overlapped with H3K27me3 from all methods to keep only these nucleosomes overlapped with H3K27me3 peaks from at least one method. With the sequence‐depth normalized bigwig files and the bed files, heatmaps were plotted using deepTools computeMatrix and plotHeatmap.

### 5.11 Analysis for Relations Between Histone Marks and Gene Expression Levels

To get the transcriptome in seedlings of Arabidopsis, we downloaded RNA‐seq data from ArrayExpress (E‐MTAB‐10965, Zhu et al. 2023). Reads were quality‐checked using fastqc (0.12.1) and trimmed with fastp (Version 0.23.4, ‐w 16 ‐l 30 ‐‐trim_poly_x ‐‐poly_x_min_len 32) (Chen et al. 2018).

We used Kallisto (Version 0.48.0) (Bray et al. 2016) to pseudo‐align the trimmed reads to the reference transcriptome. The output of Kallisto, which contains transcripts per million (TPM) numbers, was read and normalized using Sleuth (Version 0.30.1) (Pimentel et al. 2017) to obtain normalized transcriptome data. R packages dplyr (Version 1.1.4) and ggplot2 (Version 3.5.1) were used to process the data and plot transcription levels for genes marked or not marked by different histone modifications.

## Author Contributions

Y.F. performed the experiments and analyzed the data. M.W.S. contributed to data analysis and provided assistance and feedback. S.S. provided supervision, analyzed the data, and acquired funding. Y.F. and S.S. wrote the manuscript, with M.W.S. commenting on it.

## Funding

This work was supported by the University of Zurich and the Schweizerischer Nationalfonds zur Förderung der Wissenschaftlichen Forschung (SNF, to S.S.) (PR00P3_201653).

## Conflicts of Interest

The authors declare conflicts of interest.

## Peer Review

The peer review history for this article is available in the Supporting Information for this article.

## Supporting information


**Data S1:** Supporting Information.

**Table S1:** Alignment statistics of CUT&Tag libraries.
**Table S2:** Peak calling statistics of CUT&Tag libraries.
**Table S3:** i5 and i7 primers used in this study.

**Figure S1:** pld370161‐sup‐0003‐Supplementary_Information.docx. **A comparison of the performance of different H3 antibodies in CUT&Tag** Tapestation profiles show the CUT&Tag libraries generated by four different H3 antibodies. Although all the antibodies are ChIP‐grade, they show distinctly different performances in CUT&Tag. The antibody Active Motif #39064 generated libraries with much higher concentrations than libraries generated with the other antibodies. One antibody, Abcam #10799, seems to be incompatible with CUT&Tag. Each antibody has been tested with two different number of nuclei, labeled as 1× and 2×.
**Figure S2: Comparison between CUT&Tag and CUT&RUN H3K27me3 profiles** An overview of a 228‐kb genomic window, showing the similarity between the H3K27me3 profile CUT&Tag and that of a previously published CUT&RUN dataset [1].
**Figure S3: Correlation matrix of CUT&Tag, ChIP‐seq, ChIPmentation, and CUT&RUN H3K27me3 datasets and their corresponding controls.** Pearson correlation coefficients (PCCs) between every two samples are indicated. The matrix shows the H3K27me3 datasets from all methods cluster together with PCCs ranging from 0.54 to 0.83, while the controls are clustered apart.
**Figure S4: Examples of CUT&Tag‐specific and ChIP‐seq‐specific peaks.** (a) Browser views of two examples of CUT&Tag‐specific peaks on exons. (b) Browser views of two examples of intergenic ChIP‐specific peaks. Please note that because these peaks are weak, the scales used here are (0–3), which is different from the scales in other figures.
**Figure S5: Comparison of H3K27me3 signals across different GC contents between introns and exons.** The plot shows H3K27me3 log mean signal across different GC contents, from 30% to 70%, of introns and exons. The H3K27me3 signals in exons are not notably higher than those in introns.
**Figure S6: Correlation between CUT&Tag datasets of different histone modifications and the controls** A heat plot shows the correlation between replicates of different histone modifications and the H3 control. Replicates for the same histone modification always cluster together and show high correlations (Pearson correlation coefficient > 0.6), which shows the reproducibility of CUT&Tag experiments.
**Figure S7: Examples of genes with different chromatin states** Browser views showing examples of genes at different chromatin states: (a) *AP1* (*APETALA1*) and *STM* (*SHOOT MERISTEMLESS*): polycomb repression; (b) *RPL7B* (*RIBOSOMAL PROTEIN L7B*) and *UBC1* (*UBIQUITIN CARRIER PROTEIN 1*): active transcription; (c) *TCP15* (*TEOSINTE BRANCHED1/CYCLOIDEA/PCF 15*) and *ACT2* (*ACTIN 2*): mixed.
**Figure S8: Comparing the resolutions of CUT&Tag and CUT&RUN** Browser views showing H3K27me3 profiles from CUT&Tag and CUT&RUN, together with a nucleosome occupancy profile. The tracks show that the CUT&Tag H3K27me3 profile correlates well with the shape of nucleosome occupancy, while the CUT&RUN H3K27me3 profile correlates with nucleosome occupancy to a weaker degree.
**Figure S9: The preparation of pA‐Tn5 transposase complex and the tests for its enzymatic activity.** (a) The preparation process of pA‐Tn5, with the protocol from Li et al. (2021) and Henikoff et al. (2020). (b) Testing the enzyme activity of the loaded pA‐Tn5 by digesting a plasmid. As indicated by the red arrow, the plasmid was digested only in the case that pA‐Tn5 is loaded, active, and Mg^2+^ is present. (c) Comparison of home‐made pA‐Tn5 activity with a commercial pA‐Tn5 from Active Motif. The digestion results of the homemade pA‐Tn5 are comparable to those of the commercial pA‐Tn5, showing their similar activity.
**Figure S10: The comparison of performance of freshly loaded pA‐Tn5 and pA‐Tn5 that has been stored for 16 months after loading.** Tapestation profiles show the difference in performance of freshly loaded pA‐Tn5 and pA‐Tn5 that have been stored for 16 months after loading. The freshly loaded pA‐Tn5 generated libraries with nucleosome‐ladder patterns from the nuclei of *Arabidopsis* and did not produce a detectable signal when no nuclei were used. The pA‐Tn5 that have been stored for 16 months after loading generated signals without nucleosome‐ladder patterns and also generated a visible library when no nuclei were used, indicating a high level of tagmented *E. coli* DNA.

## Data Availability

Raw sequencing data are available at NCBI under the accession number PRJNA1294591. The script used for analysis is available in the GitHub repository: https://github.com/yixuan‐fu22/Arabidopsis_CUT_Tag_method_Plant_Direct
