# Supplementary material for: CUT&Tag for High‐Resolution Epigenomic Profiling From a Low Amount of Arabidopsis Tissue
Source: Plant Direct. 2026 Apr 10;10(4):e70161. doi: 10.1002/pld3.70161 (PMC13067811; doi:10.1002/pld3.70161)
Supplement: Supplementary file 3 — Figure S1: pld370161‐sup‐0003‐Supplementary_Information.docx. A comparison of the performance of different H3 antibodies in CUT&Tag Tapestation profiles show the CUT&Tag libraries generated by four different H3 antibodies. Although all the antibodies are ChIP‐grade, they show distinctly different performances in CUT&Tag. The antibody Active Motif #39064 generated libraries with much higher concentrations than libraries generated with the other antibodies. One antibody, Abcam #10799, seems to be incompatible with CUT&Tag. Each antibody has been tested with two different number of nuclei, labeled as 1× and 2×. Figure S2: Comparison between CUT&Tag and CUT&RUN H3K27me3 profiles An overview of a 228‐kb genomic window, showing the similarity between the H3K27me3 profile CUT&Tag and that of a previously published CUT&RUN dataset [1]. Figure S3: Correlation matrix of CUT&Tag, ChIP‐seq, ChIPmentation, and CUT&RUN H3K27me3 datasets and their corresponding controls. Pearson correlation coefficients (PCCs) between every two samples are indicated. The matrix shows the H3K27me3 datasets from all methods cluster together with PCCs ranging from 0.54 to 0.83, while the controls are clustered apart. Figure S4: Examples of CUT&Tag‐specific and ChIP‐seq‐specific peaks. (a) Browser views of two examples of CUT&Tag‐specific peaks on exons. (b) Browser views of two examples of intergenic ChIP‐specific peaks. Please note that because these peaks are weak, the scales used here are (0–3), which is different from the scales in other figures. Figure S5: Comparison of H3K27me3 signals across different GC contents between introns and exons. The plot shows H3K27me3 log mean signal across different GC contents, from 30% to 70%, of introns and exons. The H3K27me3 signals in exons are not notably higher than those in introns. Figure S6: Correlation between CUT&Tag datasets of different histone modifications and the controls A heat plot shows the correlation between replicates of different histone m [file PLD3-10-e70161-s003.docx]

**Supplementary Information**


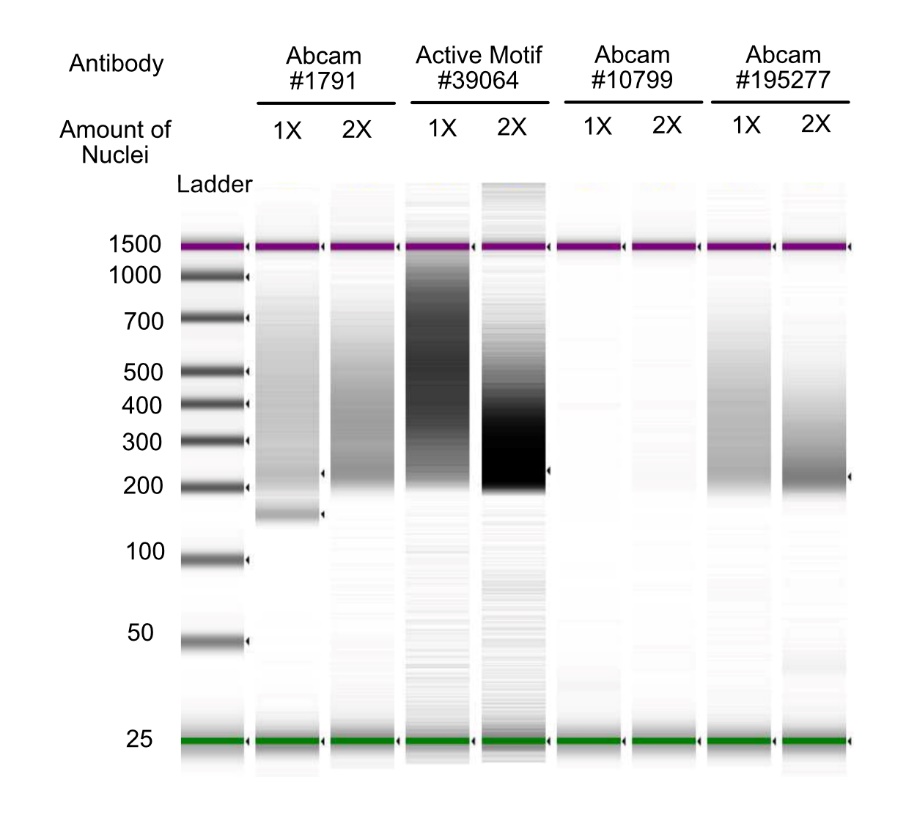


**Supplementary Figure 1. A comparison of the performance of different H3 antibodies in CUT&Tag**

Tapestation profiles show the CUT&Tag libraries generated by four different H3 antibodies. Although all the antibodies are ChIP-grade, they show distinctly different performances in CUT&Tag. The antibody Active Motif #39064 generated libraries with much higher concentrations than libraries generated with the other

antibodies. One antibody, Abcam #10799, seems to be incompatible with CUT&Tag. Each antibody has been tested with two different number of nuclei, labelled as 1X and 2X.


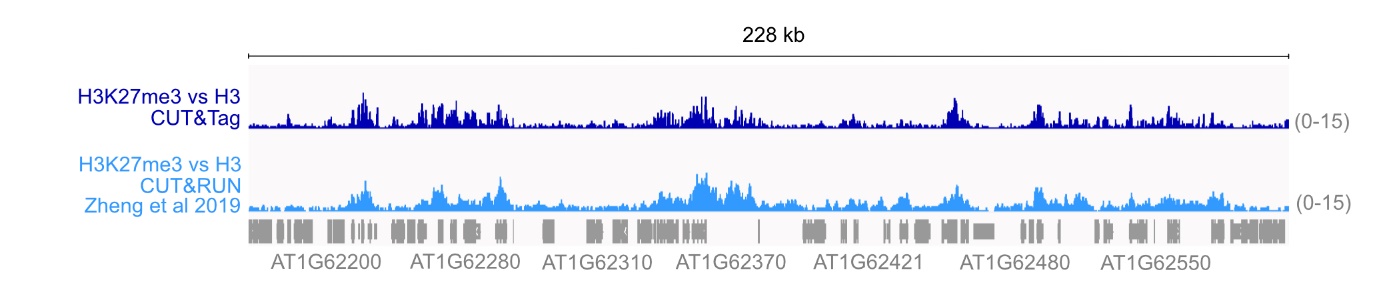


**Supplementary Figure 2. Comparison between CUT&Tag and CUT&RUN H3K27me3 profiles**

An overview of a 228 kb genomic window, showing the similarity between the H3K27me3 profile CUT&Tag and that of a previously published CUT&RUN dataset[1].

**
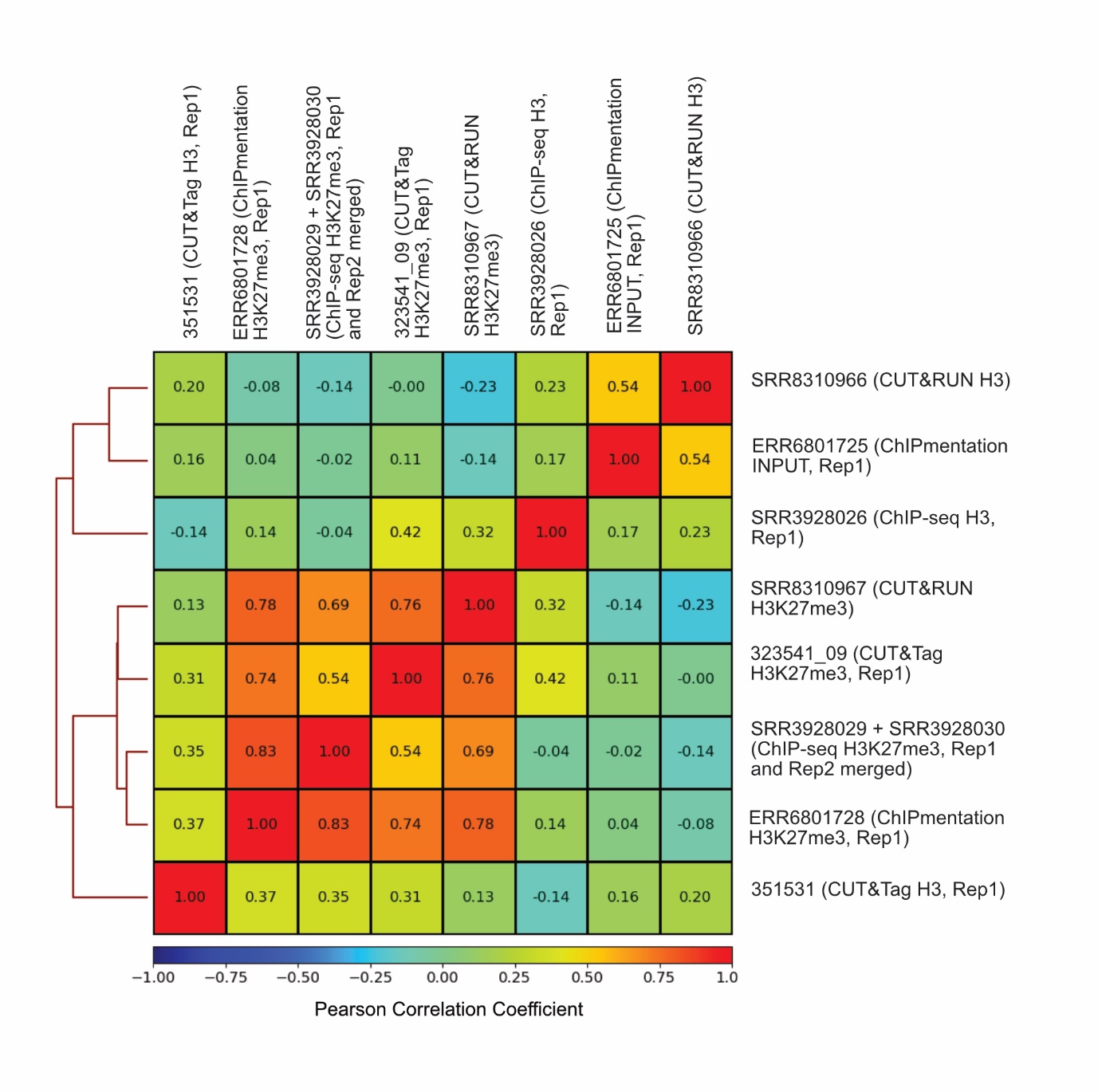
**

**Supplementary Figure 3. Correlation matrix of CUT&Tag, ChIP-seq, ChIPmentation, and CUT&RUN H3K27me3 datasets and their corresponding controls.**

Pearson Correlation Coefficients (PCCs) between every two samples are indicated. The matrix shows the H3K27me3 datasets from all methods cluster together with PCCs ranging from 0.54 to 0.83, while the controls are clustered apart.


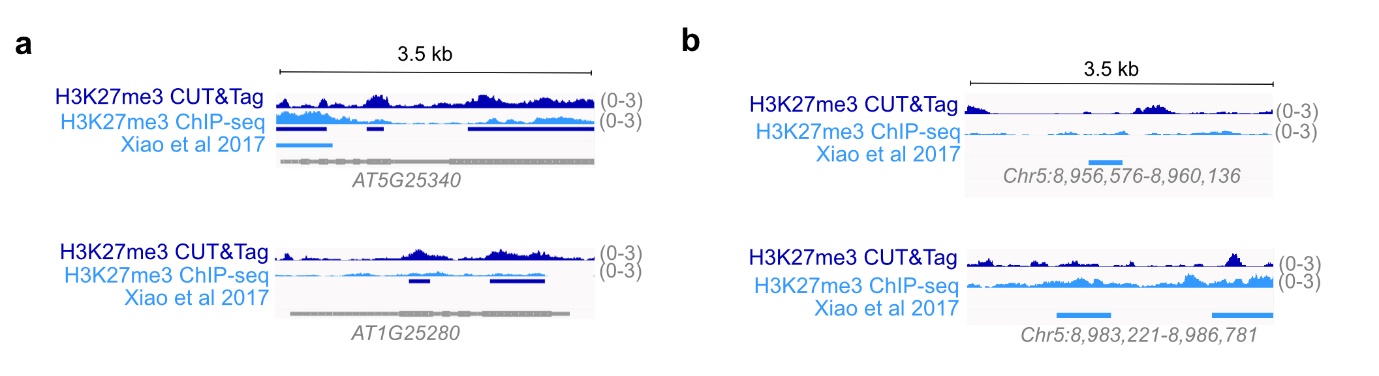


**Supplementary Figure 4. Examples of CUT&Tag-speicific and ChIP-seq-specific peaks**

(a) Browser views of two examples of CUT&Tag-specific peaks on exons. (b) Browser views of two examples of intergenic ChIP-specific peaks. Please note that because these peaks are weak, the scales used here are (0-3), which is different from the scales in other figures.

**
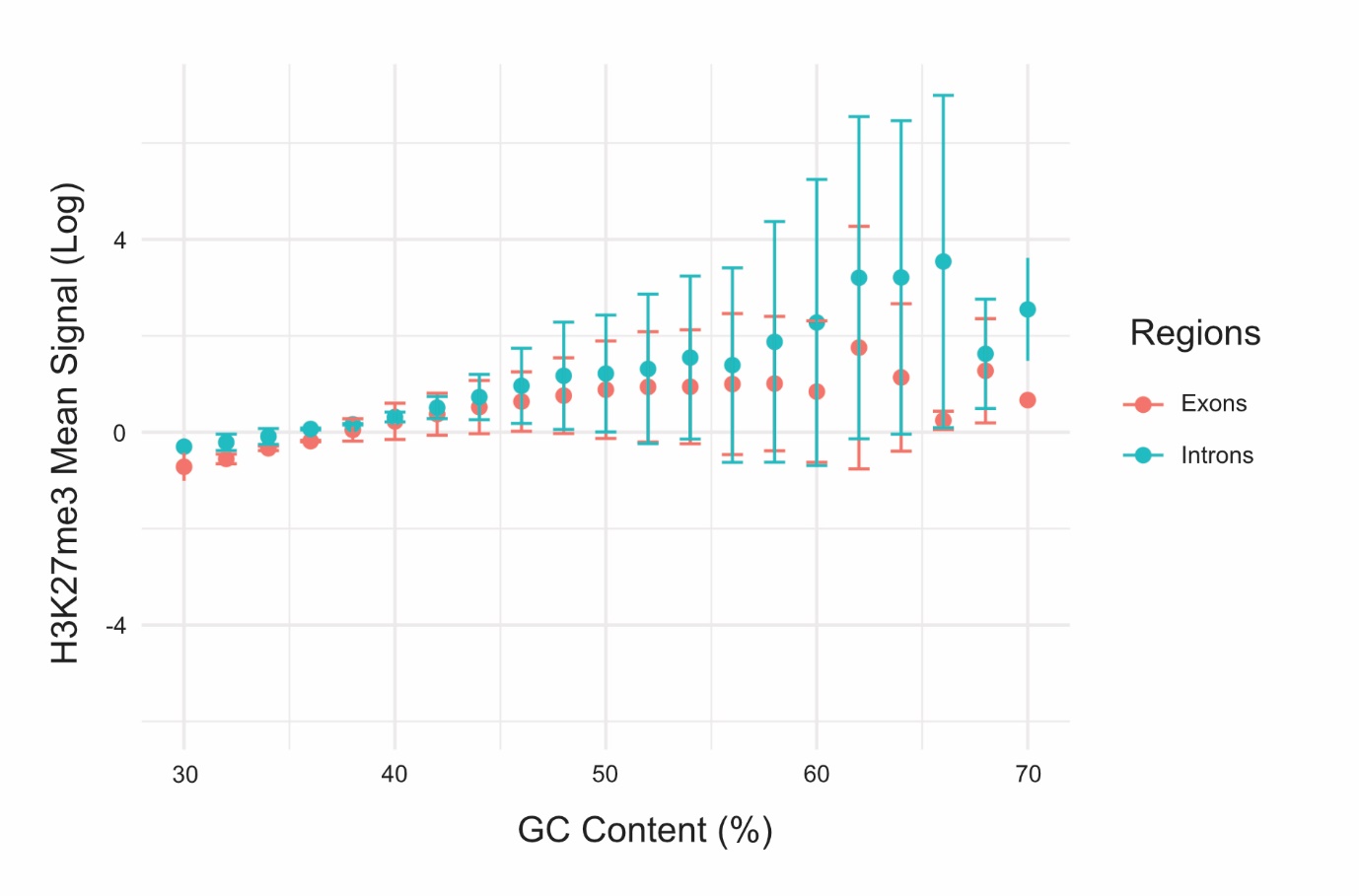
**

**Supplementary Figure 5. Comparison of H3K27me3 signals across different GC contents between introns and exons**

The plot shows H3K27me3 log mean signal across different GC contents, from 30% to 70%, of introns and exons. The H3K27me3 signals in exons are not notably higher than those in introns.


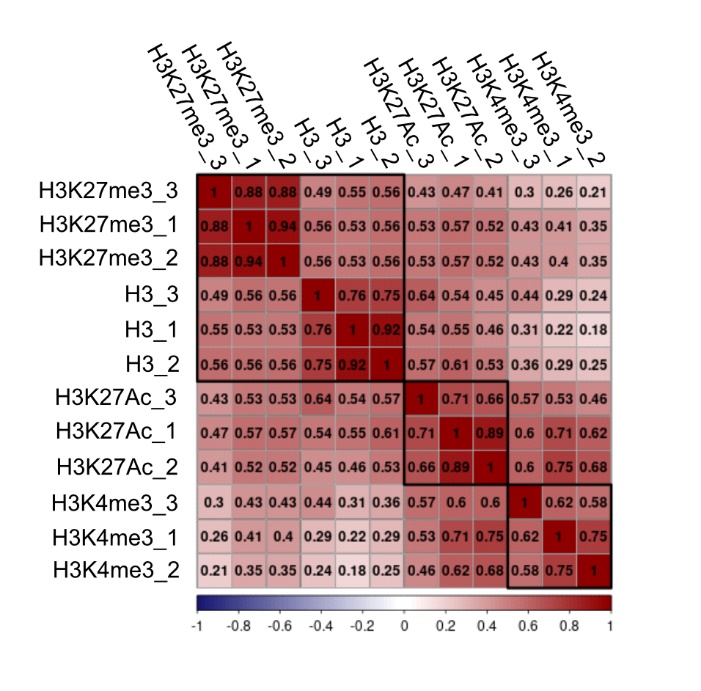


**Supplementary Figure 6. Correlation between CUT&Tag datasets of different histone modifications and the controls**

A heat plot shows the correlation between replicates of different histone modifications and the H3 control. Replicates for the same histone modification always cluster together and show high correlations (Pearson correlation coefficient > 0.6), which shows the reproducibility of CUT&Tag experiments.


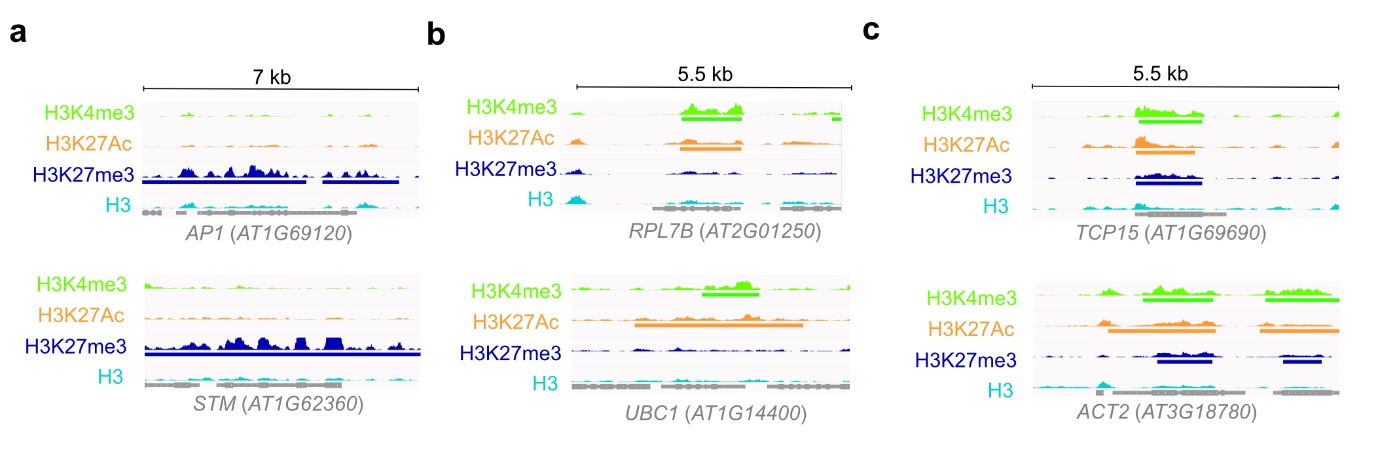


**Supplementary Figure 7. Examples of genes with different chromatin states**

Browser views showing examples of genes at different chromatin states: (a) *AP1* (*APETALA1*) and *STM* (*SHOOT MERISTEMLESS*): Polycomb repression; (b) *RPL7B* (*RIBOSOMAL PROTEIN L7B*) and *UBC1* (*UBIQUITIN CARRIER PROTEIN 1*): active transcription; (c) *TCP15* (*TEOSINTE BRANCHED1/CYCLOIDEA/PCF 15*) and *ACT2* (*ACTIN 2*): Mixed.


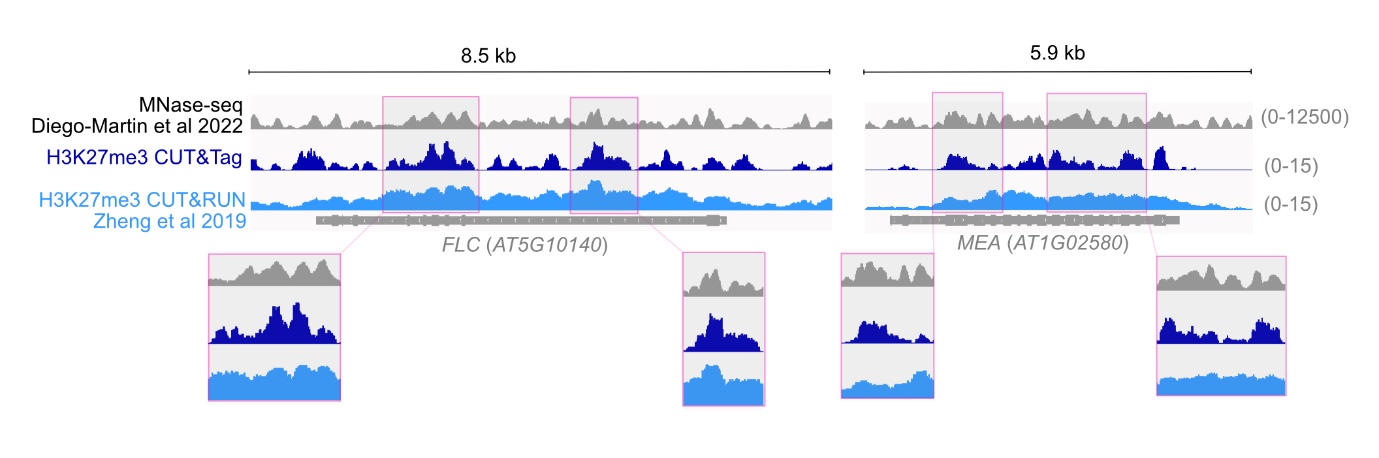


**Supplementary Figure 8. Comparing the resolutions of CUT&Tag and CUT&RUN**

Browser views showing H3K27me3 profiles from CUT&Tag and CUT&RUN, together with a nucleosome occupancy profile. The tracks show that the CUT&Tag H3K27me3 profile correlates well with the shape of nucleosome occupancy, while the CUT&RUN H3K27me3 profile correlates with nucleosome occupancy to a weaker degree.


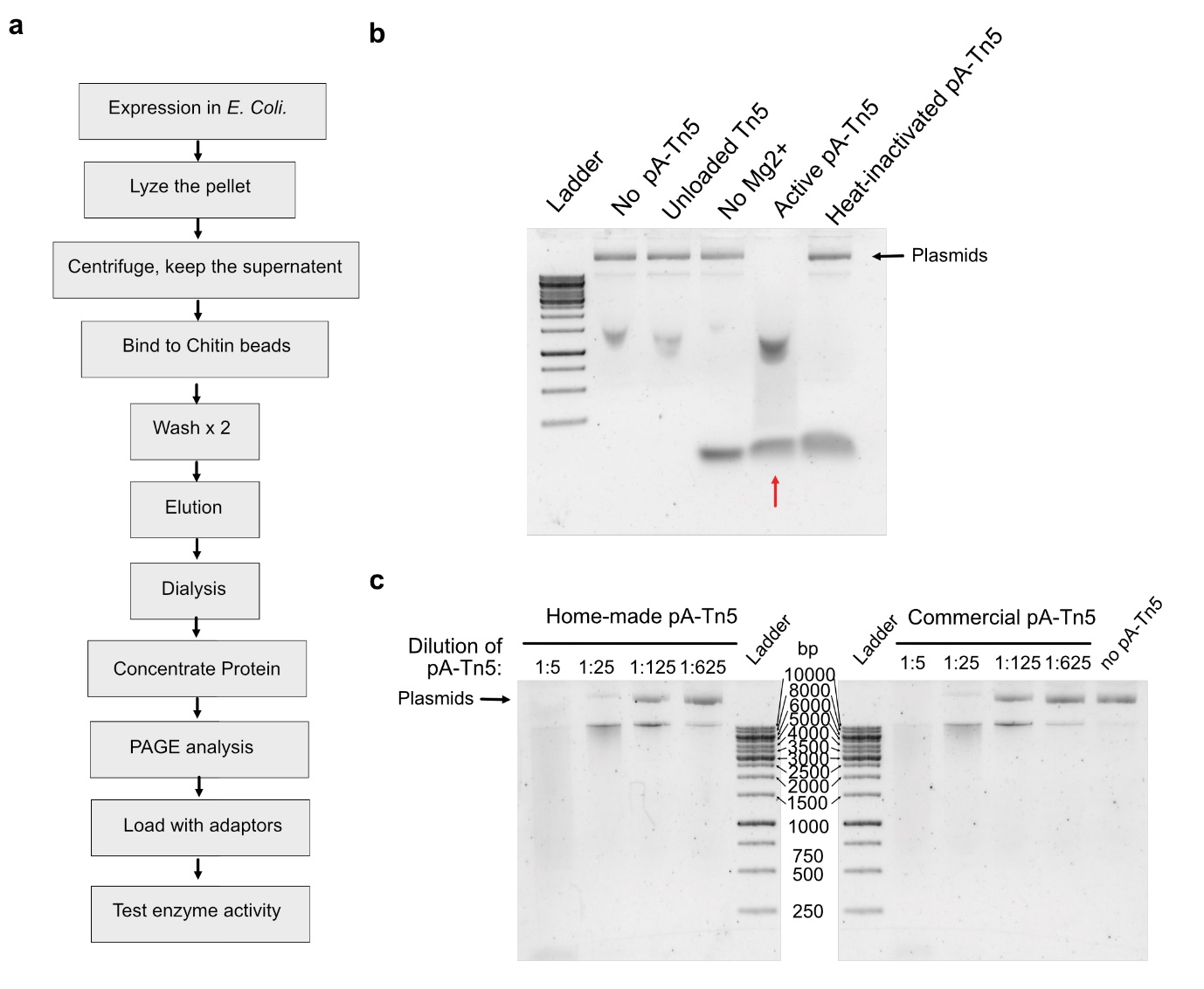


**Supplementary figure 9. The preparation of pA-Tn5 transposase complex and the tests for its enzymatic activity**

(a) The preparation process of pA-Tn5, with the protocol from Li et al. 2021 [2] and Henikoff et al. 2020 [3,4]. (b) Testing the enzyme activity of the loaded pA-Tn5 by digesting a plasmid. As indicated by the red arrow, the plasmid was digested only in the case that pA-Tn5 is loaded, active, and Mg2+ is present. (c) Comparison of home-made pA-Tn5 activity with a commercial pA-Tn5 from Active Motif. The digestion results of the homemade pA-Tn5 are comparable to those of the commercial pA-Tn5, showing their similar activity.


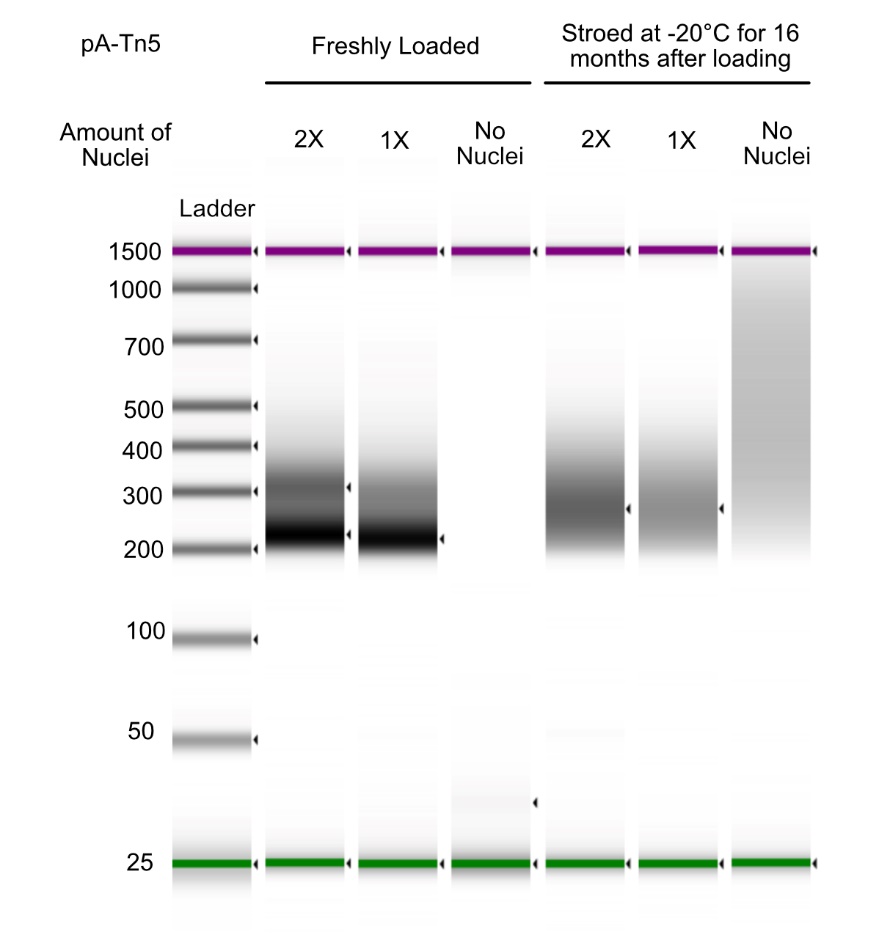


**Supplementary figure 10. The comparison of performance of freshly loaded pA-Tn5 and pA-Tn5 that has been stored for 16 months after loading**

Tapestation profiles show the difference in performance of freshly loaded pA-Tn5 and pA-Tn5 that have been stored for 16 months after loading. The freshly loaded pA-Tn5 generated libraries with nucleosome-ladder patterns from the nuclei of *Arabidopsis* and did not produce a detectable signal when no nuclei were used. The pA-Tn5 that have been stored for 16 months after loading generated signals without nucleosome-ladder patterns and also generated a visible library when no nuclei were used, indicating a high level of tagmented *E. Coli* DNA.

**Supplementary Table 1. Alignment statistics of CUT&Tag libraries**

| Number | Sample | Number of Reads Aligned to TAIR10 genome (Million Reads) | Number of Reads passed MAPQ > 10 (Miollion Reads) | Number of Reads after Deduplication (Million Reads) | Non-Redundant Fraction (NRF, = deduplicated reads / number of reads aligned, %) | Genomic with Exactly One Read Mapped (Million Reads) | Genomic with at Least One Read Mapped (Million Reads) | PCR Bottleneck Coefficient (PBC) |
| --- | --- | --- | --- | --- | --- | --- | --- | --- |
| 323541_09 | H3K27me3_1 | 32.35 | 28.59 | 17.34 | 53.60 | 4.23 | 9.70 | 0.4354 |
| 351531_10 | H3K4me3_1 | 13.85 | 12.33 | 3.45 | 24.93 | 0.60 | 2.00 | 0.3017 |
| 351531_11 | H3K27Ac_1 | 36.58 | 33.01 | 14.09 | 38.52 | 1.77 | 6.46 | 0.2737 |
| 351531_12 | H3_1 | 50.88 | 41.50 | 24.55 | 48.25 | 2.48 | 9.46 | 0.2621 |
| 323541_10 | H3K27me3_2 | 33.86 | 29.99 | 18.11 | 53.48 | 4.35 | 10.06 | 0.432 |
| 351531_22 | H3K4me3_2 | 7.35 | 6.58 | 1.87 | 25.43 | 0.50 | 1.32 | 0.3766 |
| 351531_23 | H3K27Ac_2 | 31.99 | 29.47 | 10.69 | 33.42 | 1.67 | 5.51 | 0.3025 |
| 351531_24 | H3_2 | 42.58 | 35.69 | 22.40 | 52.62 | 3.02 | 9.29 | 0.3254 |
| 351531_45 | H3K27me3_3 | 45.94 | 43.33 | 30.54 | 66.48 | 0.46 | 1.36 | 0.3372 |
| 351531_34 | H3K4me3_3 | 10.35 | 8.70 | 1.90 | 18.37 | 1.07 | 3.21 | 0.3327 |
| 351531_35 | H3K27Ac_3 | 19.16 | 16.49 | 4.89 | 25.53 | 1.46 | 4.58 | 0.3196 |
| 351531_36 | H3_3 | 36.12 | 27.81 | 8.72 | 24.15 | 1.97 | 9.82 | 0.2008 |

**Supplementary Table 2. Peak calling statistics of CUT&Tag libraries**

| Number | Sample | Control Number | Control Name | Peak Number | Average Peak Length (bp) | Percentage of Genome Covered by Peaks (%) | Fraction of Reads in Peaks (FriP, %) |
| --- | --- | --- | --- | --- | --- | --- | --- |
| 323541_09 | H3K27me3_1 | 351531_12 | H3_1 | 24824 | 615.9 | 12.80 | 46.1 |
| 351531_10 | H3K4me3_1 | 351531_12 | H3_1 | 15058 | 746.4 | 9.41 | 46.39 |
| 351531_11 | H3K27Ac_1 | 351531_12 | H3_1 | 23587 | 668.6 | 13.20 | 39.61 |
| 323541_10 | H3K27me3_2 | 351531_24 | H3_2 | 21796 | 675.6 | 12.32 | 43.91 |
| 351531_22 | H3K4me3_2 | 351531_24 | H3_2 | 11046 | 917.5 | 8.48 | 42.64 |
| 351531_23 | H3K27Ac_2 | 351531_24 | H3_2 | 18398 | 831.3 | 12.80 | 40.76 |
| 351531_45 | H3K27me3_3 | 351531_36 | H3_3 | 16702 | 857.3 | 11.98 | 46.18 |
| 351531_34 | H3K4me3_3 | 351531_36 | H3_3 | 4280 | 601.0 | 2.15 | 18.44 |
| 351531_35 | H3K27Ac_3 | 351531_36 | H3_3 | 1801 | 497.9 | 0.75 | 9.82 |

**Supplementary Table 3. i5 and i7 primers used in this study**

| **Name** | **Barcodes** | **Primer Sequence** |
| --- | --- | --- |
| C&T i5-1 | TAGATCGC | AATGATACGGCGACCACCGAGATCTACACTAGATCGCTCGTCGGCAGCGTCAGATGTGTAT |
| C&T i5-2 | CTCTCTAT | AATGATACGGCGACCACCGAGATCTACACCTCTCTATTCGTCGGCAGCGTCAGATGTGTAT |
| C&T i5-3 | TATCCTCT | AATGATACGGCGACCACCGAGATCTACACTATCCTCTTCGTCGGCAGCGTCAGATGTGTAT |
| C&T i5-4 | AGAGTAGA | AATGATACGGCGACCACCGAGATCTACACAGAGTAGATCGTCGGCAGCGTCAGATGTGTAT |
| C&T i5-5 | GTAAGGAG | AATGATACGGCGACCACCGAGATCTACACGTAAGGAGTCGTCGGCAGCGTCAGATGTGTAT |
| C&T i5-6 | ACTGCATA | AATGATACGGCGACCACCGAGATCTACACACTGCATATCGTCGGCAGCGTCAGATGTGTAT |
| C&T i5-7 | AAGGAGTA | AATGATACGGCGACCACCGAGATCTACACAAGGAGTATCGTCGGCAGCGTCAGATGTGTAT |
| C&T i5-8 | CTAAGCCT | AATGATACGGCGACCACCGAGATCTACACCTAAGCCTTCGTCGGCAGCGTCAGATGTGTAT |
| C&T i7-1 | TAAGGCGA | CAAGCAGAAGACGGCATACGAGATTCGCCTTAGTCTCGTGGGCTCGGAGATGTG |
| C&T i7-2 | CGTACTAG | CAAGCAGAAGACGGCATACGAGATCTAGTACGGTCTCGTGGGCTCGGAGATGTG |
| C&T i7-3 | AGGCAGAA | CAAGCAGAAGACGGCATACGAGATTTCTGCCTGTCTCGTGGGCTCGGAGATGTG |
| C&T i7-4 | TCCTGAGC | CAAGCAGAAGACGGCATACGAGATGCTCAGGAGTCTCGTGGGCTCGGAGATGTG |
| C&T i7-5 | GGACTCCT | CAAGCAGAAGACGGCATACGAGATAGGAGTCCGTCTCGTGGGCTCGGAGATGTG |
| C&T i7-6 | TAGGCATG | CAAGCAGAAGACGGCATACGAGATCATGCCTAGTCTCGTGGGCTCGGAGATGTG |
| C&T i7-7 | CTCTCTAC | CAAGCAGAAGACGGCATACGAGATGTAGAGAGGTCTCGTGGGCTCGGAGATGTG |
| C&T i7-8 | CAGAGAGG | CAAGCAGAAGACGGCATACGAGATCCTCTCTGGTCTCGTGGGCTCGGAGATGTG |
| C&T i7-9 | GCTACGCT | CAAGCAGAAGACGGCATACGAGATAGCGTAGCGTCTCGTGGGCTCGGAGATGTG |
| C&T i7-10 | CGAGGCTG | CAAGCAGAAGACGGCATACGAGATCAGCCTCGGTCTCGTGGGCTCGGAGATGTG |
| C&T i7-11 | AAGAGGCA | CAAGCAGAAGACGGCATACGAGATTGCCTCTTGTCTCGTGGGCTCGGAGATGTG |
| C&T i7-12 | GTAGAGGA | CAAGCAGAAGACGGCATACGAGATTCCTCTACGTCTCGTGGGCTCGGAGATGTG |
