# Supplementary material for: CUT&Tag for High‐Resolution Epigenomic Profiling From a Low Amount of Arabidopsis Tissue
Source: Plant Direct. 2026 Apr 10;10(4):e70161. doi: 10.1002/pld3.70161 (PMC13067811; doi:10.1002/pld3.70161)
Supplement: Supplementary file 1 — Data S1: Supporting Information. [file PLD3-10-e70161-s002.pdf]

CUT&Tag for high-resolution epigenomic profiling from a low amount of Arabidopsis tissue

Sara Simonini, Yixuan Fu , Dr. Marc W Schmid.

Decision letter Round 1:

May 9, 2025

Sara Simonini

Universitat Zurich

Zürich, N/A

Switzerland

RE: CUT&Tag for high-resolution epigenomic profiling from a low amount of Arabidopsis tissue

Dear Dr. Simonini:

Thank you for submitting to Plant Direct. All required reviews have been returned and we have now finished our evaluation of your manuscript. In light of the reviewers' and editor's comments, further revisions are needed before the paper can be accepted for publication in Plant Direct.

Please view the editors' and reviewers' comments below and use their suggestions as a guide while you work on your revision.

When uploading the revised version of this article, please be sure to include the following:

-A word document that contains your response to the reviewers. You should respond to each reviewer comment and note the changes made to the manuscript. If you do not agree with a reviewer's comment and choose not to make a suggested revision, please explain why. Please try to provide as complete an answer as possible to each reviewer's criticisms in the "response to reviewers" section.

-A tracked changes document

- A clean version of the latest version of the manuscript

To upload your revision, please click the link below.

https://plantdirect.msubmit.net/cgi-bin/main.plex?el=A6Lr1lx4A6pEZ3I7A9ftdxl85ARzNeI0u7gs2x6qtAQZ

In order to provide as timely a service as possible, we ask that your revision is resubmitted within three months after receipt of this request. If an extension is needed, please send a request, along with a brief explanation, to the editorial office at plantdirect@wiley.com .

Thank you very much for giving us an opportunity to review your work. I look forward to receiving the next version.

Sincerely,

Philippe Gallusci

-- Editor comments:

-- Reviewer comments:

Reviewer #1:

The authors present an optimised CUT&Tag protocol for Arabidopsis, demonstrate successful profiling of three histone marks from ~10 mg of seedlings, and benchmark the results against ChIP-seq/ChIPmentation, CUT&RUN and MNase-seq. The work fills a clear methodological gap for plant labs with limited material and, if fully documented, will be a useful resource. Data quality is promising (particularly for H3K27me3), and the comparisons are broadly convincing.

However, several issues regarding data completeness, quantitative validation, and clarity of presentation need to be addressed before the study meets Plant Direct's standards of rigour and reproducibility. I therefore recommend MAJOR REVISION.

Major Points:

Data availability

The NCBI accession is still listed as "XXXXXxxxxx". The raw FASTQ files, processed bigWig tracks and peak calls must be publicly accessible at submission.

Custom scripts/parameters (e.g. awk one-liners) should be deposited in a code repository and linked.

Replicate depth and quality metrics

Two H3K4me3 replicates and one H3K27Ac replicate yield <2 M deduplicated fragments (Suppl. Table 1). Please justify why these libraries were included and provide FRiP, NRF and PCR-bottle-neck coefficients for every sample.

Correlation heat-maps (Suppl. Fig 4) show Pearson r ≈0.6 between some replicates - low for genome-wide CUT&Tag. Include scatterplots and IDR analysis or state why IDR is inappropriate.

Resolution claims

The statement that CUT&Tag reaches "single-nucleosome" resolution is based on visual inspection (Fig 6). Provide aggregate plots of fragment centre vs MNase dyads and a quantitative peak width comparison (e.g. FWHM) with ChIP-seq.

Disentangle resolution from background smoothing: is the narrowness preserved after normalising coverage to equal read counts?

Exon enrichment of H3K27me3

The claimed exon bias (Fig 3) could arise from mappability or GC-bias differences between introns/exons. Include input-normalised control (H3 total or naked DNA tracks) and/or compute expected vs observed coverage given sequence composition.

Compare with a deeply sequenced Arabidopsis ChIP-seq H3K27me3 dataset generated from seedlings rather than leaves to rule out tissue effects.

Controls

No IgG or "no-primary" control is shown; without them it is difficult to judge antibody specificity and background. At minimum, include a no-primary CUT&Tag track in IGV screenshots and provide FRiP for it.

The manuscript notes E. coli DNA as a spike-in but does not show spike-in normalisation. Supply the normalised tracks or state that spike-in was not used and why.

Statistics and wording

Many figure legends report only "****, p{less than or equal to}

0.0001" without test details or effect sizes. Provide exact p-values and sample sizes.

Avoid categorical statements such as "CUT&Tag is unmatched by other profiling techniques" - temper with quantitative evidence.

Reviewer #2:

This study is comparing the CUT&Tag technique for epigenomic profiling to that of other techniques, with a specific emphasis on the comparison to ChIP-Seq, in Arabidopsis

thaliana. The authors showed both the success of the CUT&Tag technique and the limitations of it. Specifically, the enrichment of genes, with some loss of intergenic epigenic profiling.

Overall, I agreed with many of the authors conclusions. I think it does confirm data from other published results, which is important, and is a good study with good information for others wanting to use this technique.

Decision letter Round 2:

March 4, 2026

Sara Simonini

Universitat Zurich

Zürich, N/A

Switzerland

RE: CUT&Tag for high-resolution epigenomic profiling from a low amount of Arabidopsis tissue

Dear Sara:

I am sincerely sorry for the long delay with your manuscript. I have now taken over handling this and please see the brief comments below from reviewers. I would like to give you the opportunity to make any final amendments in light of those points and resubmit your revision, though I anticipate minimal change given our timeline. No further review is needed - we will then be able to move to publication.

When uploading the revised version of this article, please be sure to include the following:

-A word document that contains your response to the reviewers. You should respond to each reviewer comment and note the changes made to the manuscript. If you do not agree with a reviewer's comment and choose not to make a suggested revision, please explain why. Please try to provide as complete an answer as possible to each reviewer's criticisms.

-A tracked changes document with each change highlighted

- A clean version of the latest version of the manuscript

To upload your revision, please click the link below.

https://plantdirect.msubmit.net/cgi-bin/main.plex?el=A4Lr7lx6B1pEZ1I2A9ftdxl85ARzNeI0u7gs2x6qtAQZ

Please resubmit at your earliest convenience.

Please note that, in addition to publishing reviewer comments, the author's responses to review comments will also be published alongside the final version of the paper. If you would not like the author's responses to be published, please contact the editorial office at plantdirect@wiley.com .

I look forward to receiving your final version very soon.

Regards

Jonathan Ingram

Editor, Plant Direct

-- Reviewer comments:

Reviewer #2:

The present manuscript describes the technique "CUT&Tag" for Arabidopsis and compares their results from other peer reviewed and published datasets generated by similar techniques. They showed comparable results, but by using less starting material. Overall, I think this is a solid study that validates other datasets and describes a protocol that others might find valuable.

It might be of interest to the authors to include in the background that Cut&Tag has been used in other plants, particularly Rice, and maybe how this study expands, or builds upon, or is distinct from those. I will admit that an Arabidopsis specific protocol, as presented here, is probably sufficient. Thought I would make that suggestion.

Second, I think it would be interesting to compare the histone profile that was generated from this study with DNA methylation datasets, as there is some known correlation and anti-correlations between the two. I will also admit that this comparison might be beyond the scope of this manuscript. Just a thought I would share.

Reviewer #3:

The authors present a CUT&Tag protocol for analysing histone modification in Arabidopsis tissues, going down to ca 10 mg input material. This is a revised version of the manuscript that in my opinion has reflected on the main concerns of the reviewer 1, which were mostly related to the data analyses. In their responses, it would have been helpful if the authors had provided reference to the concrete Figures and changes made. Overall, however, I find that the establishment and detailed description of the CUT&Tag protocol adapted for plant tissues is a valuable resource for the community. Although there are patterns in the observed signal distribution (e.g. exon-biased distribution of H3K27me3) that will need further study, making such protocol available is a valuable opportunity to generate further datasets to understand these patterns.

I have several minor comments:

- Optimization of H3 antibody is shown in Supplementary Fig.1 - have the other used histone modification antibodies been also validated for use in CUT&Tag?

- Following up on the discussion with reviewer 1, it should be better specified which analyses (figures) use normalized signals (by H3) and which do not - i.e. are the identified peaks (and associated loci) coverage or enrichment peaks?

- Line 278: the G-quadruplex-forming sequence seems to be a complement to the telobox motif? Or is this indeed a separate motif?

- Line 877: Supplementary Figure 1: legend: it is not clear what nuclei 1x or 2x indicates. Please specify in the legend

- Please proofread the manuscript carefully for typos: e.g.

Line 31 - "In the end, we present..." instead of "presented"

Line 307: typo - correlation

Line 434: typo - "culture"

Line 887: Supplementary Figure 1: legend title- typo: ..."CUT&Tag-SPECIFIC"

Line 915: typo: "FROM Active Motif..."

Decision letter Round 3:

March 12, 2026

Sara Simonini

Universitat Zurich

Zürich, N/A

Switzerland

MSID: 2024-01679R2

MS TITLE: CUT&Tag for high-resolution epigenomic profiling from a low amount of Arabidopsis tissue

Dear Sara:

I am pleased to inform you that your manuscript "CUT&Tag for high-resolution epigenomic profiling from a low amount of Arabidopsis tissue" has been accepted for publication in Plant Direct.

Your article will appear online in the next available issue of Plant Direct. To ensure your article gets published as quickly as possible, please pay attention to the steps detailed below. We have found that most of the delays happen at this stage, especially at the payment stage, so please respond as quickly as possible when prompted.

License Agreement: Once your article has been accepted it will move to Production and undergo admin and file checking - you may receive an email with any queries we have at this stage. When all required items are received by the publisher and queries resolved, the corresponding author will receive an email from Wiley's Author Services system which will ask them to log on at https://authorservices.wiley.com/bauthor and will present them with the appropriate license for completion. Your article cannot be published until both the signed license agreement and payment of the article fee have been received.

Payment of the Open Access Article Publication Fee: All articles published in Plant Direct are fully open access: immediately and freely available to read, download and share. Plant Direct charges a publication fee to cover the publication costs. The corresponding author for this manuscript should have already received a quote from the payments team (cs-openaccess@wiley.com) with the estimated article publication fee; please email cs-openaccess@wiley.com if this has not been received. The corresponding author should log on to the Wiley Author Services site, where the publication fee can be paid by credit card or an invoice. Pro Forma can also be requested. Payment of the publication charge must be received before the article will be published online.

Proofs: You will have the opportunity to look over your paper once more when you receive the author proofs for your article. The proofs will be with you in approximately two weeks. Please note that, in addition to publishing reviewer comments, the author's responses to review comments will also be published alongside the final version of the paper. If you would not like the author's responses to be published, please contact the editorial office at plantdirect@wiley.com .

Promotion of your article: You can help your research get the attention it deserves! Check out Wiley's free Promotion Guide for best-practice recommendations for promoting your work at www.wileyauthors.com/eeo/guide. And learn more about Wiley Editing Services which offers professional video, design, and writing services to create shareable video abstracts, infographics, conference posters, lay summaries, and research news stories for your research at www.wileyauthors.com/eeo/promotion.

Thank you again for your contribution to Plant Direct. If you have any questions, feel free to contact the editorial office at plantdirect@wiley.com .

Sincerely,

Jonathan Ingram

Editor, Plant Direct

Response letter Round 1:

We would like to thank the reviewers for their positive assessment, constructive comments, and helpful suggestions, as well as for identifying errors in our manuscript. The review process has significantly improved the quality of our work. We have addressed all the points raised and provide a point-by-point response to each of the reviewers' comments below.

Reviewer #1:

The authors present an optimised CUT&Tag protocol for Arabidopsis, demonstrate successful profiling of three histone marks from ~10 mg of seedlings, and benchmark the results against ChIP-seq/ChIPmentation, CUT&RUN and MNase-seq. The work fills a clear methodological gap for plant labs with limited material and, if fully documented, will be a useful resource. Data quality is promising (particularly for H3K27me3), and the comparisons are broadly convincing.

However, several issues regarding data completeness, quantitative validation, and clarity of presentation need to be addressed before the study meets Plant Direct's standards of rigour and reproducibility. I therefore recommend MAJOR REVISION.

Major Points:

Data availability

The NCBI accession is still listed as "XXXXXxxxxx". The raw FASTQ files, processed bigWig tracks and peak calls must be publicly accessible at submission.

Custom scripts/parameters (e.g. awk one-liners) should be deposited in a code repository and linked.

We thank the reviewer for spotting this error. We indeed have uploaded the dataset prior to the submission of this work, but forgot to incorporate the accession number into the manuscript. We apologize for this mistake. The NCBI accession number is PRJNA1294591, and data can be downloaded here: https://dataview.ncbi.nlm.nih.gov/object/PRJNA1294591?reviewer=skaafa9v245vk3v53bup5sbpc0). These information have now been added to the manuscript.

Replicate depth and quality metrics

Two H3K4me3 replicates and one H3K27Ac replicate yield <2 M deduplicated fragments (Suppl. Table 1). Please justify why these libraries were included and provide FRiP, NRF and PCR-bottle-neck coefficients for every sample.

Correlation heat-maps (Suppl. Fig 4) show Pearson r ≈0.6 between some replicates - low for genome-wide CUT&Tag. Include scatterplots and IDR analysis or state why IDR is inappropriate.

Following the reviewer’s suggestion, we have now prepared new Supp table 1 (Alignment statistics) and Supplementary Table 2, which include FRiP, NRF, and PCR-bottle-neck coefficients.

The H3K4me3 and H3K27Ac libraries have lower numbers of reads, because these libraries were made with a batch of home-made pA-Tn5 enzyme that had partially lost activity during storage (we discussed this important aspect in the method session and in the Supplementary Figure 8). Despite this inconvenience, the quality of two replicates out of three (rep 1 and rep 2) proved to be of sufficient quality and depth for peak calling, supported by their FRiP score around or above 40%, percentages of genome covered by peaks around 10%. We’ve also presented additional validations in figure 4, including IGV screenshots, heatmap around gene bodies, and genomic feature distribution on peaks.

Replicate 3 of both H3K27Ac and H3K4me3 libraries showed lower data quality, with FRiP scores of 9.82% and 18.44%, respectively, and a reduced percentage of genome covered by peaks (0.75% for H3K27Ac and 2.15% for H3K4me3). Due to this suboptimal quality, these libraries would not be suitable for analyses that rely on individual replicates for statistical power, such as differential enrichment analysis.

However, the primary aim of our study is to demonstrate that CUT&Tag is an easy and reliable technique, and to share our optimized protocol with the plant research community. Therefore, we did not perform deeper analyses (e.g., differential peak analysis), where the inclusion of a lower-quality replicate would have had a significant negative impact.

Importantly, despite relatively low correlation scores (around 0.6), replicate 3 still clustered with the other replicates of the same histone mark and showed comparable broad signal profiles upon visual inspection. For these reasons, we retained replicate 3 for genome browser track visualization and for generating the master peak list, in which peaks are included if supported by at least two replicates. We have enriched the text with this information (second paragraph of the discussion).

Regarding the reason why we did not use the Irreproducibility Discovery Rate (IDR): we gathered from community discussions that IDR is not the best approach for broad peaks calling for histone modifications, which have a smoother dynamic range compared to that of peaks from transcription factors (source: https://github.com/ENCODE-DCC/chip-seq-pipeline2/issues/30). Therefore, we chose to adopt the approach of consensus voting (reference [47]).

Resolution claims

The statement that CUT&Tag reaches "single-nucleosome" resolution is based on visual inspection (Fig 6). Provide aggregate plots of fragment centre vs MNase dyads and a quantitative peak width comparison (e.g. FWHM) with ChIP-seq.

Disentangle resolution from background smoothing: is the narrowness preserved after normalising coverage to equal read counts?

As suggested by the Reviewer, we added new heatmaps around nucleosomes and FWHM to the script.

From the heatmaps and FWHM, the CUT&Tag data shares similarity to ChIPmentation data: both techniques reveal an enrichment of H3K27me3 and H3 signal on nucleosomes, while this enrichment is obscured in ChIP-seq and CUT&RUN data. Therefore, we conclude that CUT&Tag, similar to ChIPmentation, has a higher resolution compared to ChIP-seq and CUT&RUN. We have updated the wording accordingly.

Is the narrowness preserved after normalising coverage to equal read counts? – The read counts of replicates from CUT&Tag, ChIP-seq, and ChIPmentation we used for the heatplots are similar to each other: 17.3 M for CUT&Tag, 16.8 M for ChIPmentation, and 18.4 M for ChIP-seq (we merged two replicates of ChIP-seq H3K27me3 to reach this read number). When plotting heatmap, all samples have been normalized to reads per genome coverage (RPGC). Therefore, the effect of read counts could be excluded. We have added the information above to the method session (subsession: Comparison of CUT&Tag with ChIP-seq, ChIPmentation, CUT&RUN, and MNase datasets).

Exon enrichment of H3K27me3

The claimed exon bias (Fig 3) could arise from mappability or GC-bias differences between introns/exons. Include input-normalised control (H3 total or naked DNA tracks) and/or compute expected vs observed coverage given sequence composition.

Compare with a deeply sequenced Arabidopsis ChIP-seq H3K27me3 dataset generated from seedlings rather than leaves to rule out tissue effects.

We acknowledge that differences in mappability or GC content between introns and exons could introduce bias in the signal. To further investigate this, we have added a new Supplementary Figure (the new Supplementary Figure 5) comparing CUT&Tag signal over exons and introns. In fact, we cannot exclude the possibility that the higher degree of enrichment on exons observed in CUT&Tag data is due to GC-content difference between exons and introns, because at the same GC content, the signals between exons and introns are similar. However, the difference should not be a result of different mappability, since CUT&Tag H3K27me3 libraries have mapping rates > 95%. We reasoned that the difference between exons and introns is possibly due to the difference in GC content. However, the improved resolution of CUT&Tag compared to ChIP-seq allowed to better reveal this difference. We have updated the text and discussed this issue in the discussion accordingly.

Controls

No IgG or "no-primary" control is shown; without them it is difficult to judge antibody specificity and background. At minimum, include a no-primary CUT&Tag track in IGV screenshots and provide FRiP for it.

The manuscript notes E. coli DNA as a spike-in but does not show spike-in normalisation. Supply the normalised tracks or state that spike-in was not used and why.

We acknowledge that the way we presented the data may not have been accessible enough to the reader. In Figure 4a we have included the H3 track together with the histone modifications. The other genomic tracks in the main plots have been normalized by the corresponding controls. We have relabelled the plots to make them clear for readers.

The reason spike-in normalization was not used is primarily technical. We used two batches of pA-Tn5, which were loaded at different times and inherently contain varying amounts of E. coli DNA, which renders spike-in normalization unreliable.

Moreover, all our samples come from the same tissue type (seedlings) and genotype (Col-0), and we do not perform comparisons across tissues or genotypes. Therefore, spike-in normalization would offer limited added value in this context, and normalization based on sequencing depth is sufficient for our analyses.

To avoid potential confusion, we have removed the original statement from the manuscript.

Statistics and wording

Many figure legends report only "****, p{less than or equal to}

0.0001" without test details or effect sizes. Provide exact p-values and sample sizes.

Avoid categorical statements such as "CUT&Tag is unmatched by other profiling techniques" - temper with quantitative evidence.

We thank the reviewer for spotting this error. The p values have now been added to the plots, together with the test method and sample size. We have also revised the wording to avoid categorical statements.

Reviewer #2:

This study is comparing the CUT&Tag technique for epigenomic profiling to that of other techniques, with a specific emphasis on the comparison to ChIP-Seq, in Arabidopsis thaliana. The authors showed both the success of the CUT&Tag technique and the

limitations of it. Specifically, the enrichment of genes, with some loss of intergenic epigenic profiling.

Overall, I agreed with many of the authors conclusions. I think it does confirm data from other published results, which is important, and is a good study with good information for others wanting to use this technique.

We thank the reviewer for the supportive feedback.

Response letter Round 2:

Dear Editor,

First of all, we would like to thank you for your support of our manuscript and for handling the review process. We also thank the reviewers for their careful evaluation of our work and for their constructive comments.

Below we provide a point-by-point response to the issues raised by the reviewers.

We have revised the manuscript accordingly, and all changes are indicated in the revised version.

Reviewer #2:

The present manuscript describes the technique "CUT&Tag" for Arabidopsis and compares their results from other peer reviewed and published datasets generated by similar techniques. They showed comparable results, but by using less starting material. Overall, I think this is a solid study that validates other datasets and describes a protocol that others might find valuable.

It might be of interest to the authors to include in the background that Cut&Tag has been used in other plants, particularly Rice, and maybe how this study expands, or builds upon, or is distinct from those. I will admit that an Arabidopsis specific protocol, as presented here, is probably sufficient. Thought I would make that suggestion.

We thank the reviewer for this helpful suggestion. We have revised the Introduction

to acknowledge recent studies applying CUT&Tag in plant systems, including rice and other species. We now explicitly reference these works and clarify how our study complements these efforts by providing a validated CUT&Tag protocol optimized for Arabidopsis tissue using very low input material.

Second, I think it would be interesting to compare the histone profile that was generated from this study with DNA methylation datasets, as there is some known correlation and anti-correlations between the two. I will also admit that this comparison might be beyond the scope of this manuscript. Just a thought I would share.

We thank the reviewer for this interesting suggestion. Indeed, relationships between histone modifications and DNA methylation represent an important aspect of chromatin regulation. However, a systematic comparison with DNA methylation datasets would require additional analyses beyond the methodological scope of the present study, which focuses primarily on establishing and validating a robust CUT&Tag protocol for low-input Arabidopsis tissue. We therefore believe that such analyses would be better addressed in future studies specifically aimed at dissecting chromatin state interactions during plant development.

Reviewer #3:

The authors present a CUT&Tag protocol for analysing histone modification in Arabidopsis tissues, going down to ca 10 mg input material. This is a revised version of the manuscript that in my opinion has reflected on the main concerns of the reviewer 1, which were mostly related to the data analyses. In their responses, it would have been helpful if the authors had provided reference to the concrete Figures and changes made. Overall, however, I find that the establishment and detailed description of the CUT&Tag protocol adapted for plant tissues is a valuable resource for the community. Although there are patterns in the observed signal distribution (e.g. exon-biased distribution of H3K27me3) that will need further study, making such protocol available is a valuable opportunity to generate further datasets

to understand these patterns.

We thank the reviewer for the positive evaluation of our work and for recognizing the value of establishing and describing a CUT&Tag protocol optimized for plant tissues.

I have several minor comments:

- Optimization of H3 antibody is shown in Supplementary Fig.1 - have the other used histone modification antibodies been also validated for use in CUT&Tag?

We thank the reviewer for raising this point. To clarify this aspect, we have now added Table 1 in the main text (first Results section), listing the antibodies that we have tested and validated for CUT&Tag and that consistently produced high-quality libraries. These include antibodies recognizing H3K27me3, H3K4me3, H3K27ac, H3K4me2 and H2Aub, in addition to total H3. The table specifies the antibody source, catalogue number and host species. This addition clarifies which antibodies have been successfully validated in our experimental setup up to date.

- Following up on the discussion with reviewer 1, it should be better specified which analyses (figures) use normalized signals (by H3) and which do not - i.e. are the identified peaks (and associated loci) coverage or enrichment peaks?

We thank the reviewer for this suggestion. We have now clarified in the manuscript which analyses use H3-normalized signals and which do not. This information has been added either in the relevant figure legends or in the main text. For clarity:

- IGV browser screenshots in Fig. 2a, 2c, and 3b are H3-normalized (now explicitly labelled in the figure).
- Fig. 4a shows non-normalized signal, with H3 signal displayed in parallel for comparison.
- Heatmaps in Fig. 3c and 4b are not normalized by H3, and the corresponding H3 signals are shown alongside for reference.
- All peak-based analyses were performed using peaks called with H3 normalization.

These clarifications have been incorporated into the manuscript to avoid ambiguity.

- Line 278: the G-quadruplex-forming sequence seems to be a complement to the telobox motif? Or is this indeed a separate motif?

The reveiwer is right that the G-quadruplex-forming sequence is indeed a Telobox motif. Thank you for spotting this mistake. We have removed this motif from the Figure.

- Line 877: Supplementary Figure 1: legend: it is not clear what nuclei 1x or 2x indicates. Please specify in the legend

"1X" and "2X" refer to the quantity of nuclei used per reaction. We acknowledge that this was not clearly explained in the previous version of the manuscript. The legend of Supplementary Figure 1 has now been revised to explicitly clarify the meaning of the "1×" and "2×" nuclei conditions.

- Please proofread the manuscript carefully for typos: e.g.

Line 31 - "In the end, we present..." instead of "presented"

Line 307: typo - correlation

Line 434: typo - "culture"

Line 887: Supplementary Figure 1: legend title- typo: ..."CUT&Tag-SPECIFIC"

Line 915: typo: "FROM Active Motif..."

Typos have been corrected, thanks for spotting these errors.
